# Dual Isotope Analysis Reveals Phylogenetic Patterns and Novel Insights Into Methoxy Group Synthesis of Structural Biomolecules in Leaf and Woody Plant Tissues

**DOI:** 10.1111/pce.70134

**Published:** 2025-08-25

**Authors:** Anna Wieland, Philipp Schuler, Matthias Saurer, Valentina Vitali, Markus Greule, Frank Keppler, Marco M. Lehmann

**Affiliations:** ^1^ Institute of Earth Sciences Heidelberg University Heidelberg Germany; ^2^ School of Architecture, Civil and Environmental Engineering Ecole Polytechnique Federale de Lausanne Lausanne Switzerland; ^3^ Community Ecology, Swiss Federal Institute for Forest, Snow and Landscape Research Lausanne Switzerland; ^4^ Forest Dynamics, Swiss Federal Institute for Forest, Snow and Landscape Research WSL Birmensdorf Switzerland; ^5^ Department of Environmental Systems Science Forest Ecology, Institute of Terrestrial Ecosystems, ETH Zürich Zürich Switzerland; ^6^ Heidelberg Center for the Environment (HCE) Heidelberg University Heidelberg Germany; ^7^ Forest Soils and Biogeochemistry, Swiss Federal Institute for Forest, Snow and Landscape Research WSL Birmensdorf Switzerland

**Keywords:** carbon isotopes, hydrogen isotopes, metabolic processes, phylogenetic tree, proxy

## Abstract

Stable carbon and hydrogen isotopes of wood methoxy groups (*δ* ^2^H_meth_, *δ* ^13^C_meth_), mainly sourced by structural biomolecules like lignin and pectin, provide important insights into climatic, hydrological and physiological conditions. This study systematically investigated species‐specific *δ* ^2^H_meth_ and *δ* ^13^C_meth_ variations in leaves and woody twigs of 65 different tree species grown in a common garden. Significant phylogenetic patterns were observed in *δ* ^2^H_meth_ and *δ* ^13^C_meth_ of both tissues, with stronger signals in leaves and the most pronounced differences between angiosperms and gymnosperms. *δ* ^13^C_meth_ variations are likely explained by anatomical and physiological differences between seed types, while *δ* ^2^H_meth_ variations were attributed to temporal differences in water uptake or isotope fractionation processes. Notably, *δ* ^13^C_meth_ values were more negative in leaves than in twigs, while *δ* ^2^H_meth_ values showed no tissue‐specific difference. This suggests that serine, a methoxy precursor, is differently synthesised in autotrophic than in heterotrophic tissues. Hydrogen isotope fractionation between xylem water and twig methoxy groups averaged at −197 mUr, with mean isotope fractionation of gymnosperms −209 mUr being significantly different to that of angiosperms −184 mUr. Weak relationships between *δ* ^2^H_meth_ and *δ* ^2^H values of carbohydrates indicated that distinct signals are preserved within the two compounds. This study highlights the importance of phylogenetic considerations when using methoxy group isotopes as proxies and provides new insights into methoxy group biosynthesis.

## Summary Statement

Phylogenetic patterns were found in hydrogen and carbon isotopes of twig and leaf methoxy groups primarily at the seed type level. Strong offsets between leaves and twigs in carbon but not in hydrogen isotopes showed that methoxy groups are partly influenced by different precursors in plant tissues.

## 1 Introduction

Over the past two decades, stable hydrogen and carbon isotope measurements of wood methoxy groups (OCH_3_; *δ* ^2^H_meth_ and *δ* ^13^C_meth_) have been utilised as reliable climate proxies, offering a less labour‐intensive and more time‐efficient alternative to traditional tree ring cellulose stable isotope measurements (Keppler et al. 2004, 2007; Greule et al. 2008, 2009, 2021). Wood methoxy groups predominantly originate from lignin (ether bounded) or pectin (ester bounded), with lignin being a major tissue of woody material (25%–30%) and methoxy groups making 15%–20% of lignin polymers (Keppler et al. 2007). The pectin methoxy group content in woody material is secondary, whereas in leaves pectin can be up to 35% of the cell wall material (Keppler et al. 2004).

In the synthesis of methoxy groups, two out of three hydrogens and the carbon of the methoxy group originate from the methylene group (CH_2_) of the serine (C_3_H_7_NO_3_) C3 position. Serine has been shown to be a product of three different pathways including:

1. The glycolate pathway: part of the photorespiration and considered to be the most important pathway in photosynthetic organs (Douce et al. 2001; Ros et al. 2014). It takes place in the mitochondria matrix, where serine is formed from two glycine molecules (Watanabe et al. 2021).
2. The glycerate pathway: reverse process of a segment of the photorespiratory cycle by converting 3‐PGA via glycerate to serine either in the cytosol or peroxisomes (Kleczkowski and Givan 1988).
3. The phosphorylated pathway (PPLP): synthesis of serine in the plastids from 3‐PGA via 3‐phosphohydroxypyruvate and 3‐phosphoserine (Pizer 1963). This pathway was recently found to be more important for plant growth than the photorespiration pathways (Zimmermann et al. 2021).

Serine plays an important role in all living organisms, being an essential component of proteins and necessary for various cellular functions. It is involved in key processes like folate metabolism, synthesis of nucleotides, amino acids, phosphor lipids and sphingolipids (Kalhan and Hanson 2012; Ros et al. 2014; Zimmermann et al. 2021). It can be easily transported through the phloem (Riens et al. 1991; Ros et al. 2014), allowing serine to be synthesised via photorespiration or photosynthesis in leaves and supplied to other plant organs. However, heterotrophic tissues have been suggested to synthesise serine via the PPLP, as PPLP genes are expressed in the vasculature, shoot and root‐apical meristem, embryos, anthers, stigma and pollen grains (Benstein et al. 2013; Cascales‐Minana et al. 2013; Toujani et al. 2013; M. Wang et al. 2024). While leaves are the primary photosynthetic organs in woody plants, active chloroplasts have also been found in twigs, branches and even stems, making these tissues to non‐foliar CO_2_ fixators (Nilsen 1995; Pfanz 2007; Natale et al. 2023). Stem photosynthesis utilises CO_2_ from the atmosphere and re‐fixes internal CO_2_ released by respiration from surrounding heterotrophic tissues (Ávila et al. 2014). As CO_2_ accumulation under the bark can reach concentrations up to 1%–26%, there is sufficient CO_2_ for photosynthesis while photorespiration is limited (Cernusak and Marshall 2000; Cernusak et al. 2001; Teskey et al. 2008). However, due to significant species‐specific heterogeneity and relatively limited research on this topic, the functional features are still poorly understood, it remains unknown whether there is a systematic difference between leaf‐shedding deciduous trees and evergreen conifers (Aschan et al. 2001; Pfanz 2007; Natale et al. 2023).

During methoxy group synthesis, the methylene group (CH_2_) from serine is transferred to tetrahydrofolate (THF), forming 5,10‐CH_2_‐THF and further reduced to a methyl group (CH_3_) to from 5‐CH_3_‐THF by the flavoprotein methylenetetrahydrofolate reductase (Schmidt and Kexel 1998; Roje et al. 1999; Schmidt et al. 2003, 2006). This flavoprotein accepts reducing equivalents from NADH and transfers them to CH_2_‐THF (Trimmer 2013). It is important to note, that in plants the 5,10‐CH_2_‐THF reductase is NADH, not NADPH dependent as it is in other eukaryotes (Roje et al. 1999). The NADH pools are cell‐specific with different sources such as (a) the photosynthesis or (b) the pentose phosphate pathway, and the hydrogen transfer from these different sources is likely accompanied by individual isotopic effects (Schmidt et al. 2003; Zhang et al. 2009). Notably, NADH from photosynthesis has been observed to be substantially depleted in ^2^H (by more than 100‰) compared to that from the pentose phosphate pathway (Luo et al. 1991; Schmidt et al. 2003). During lignin synthesis, the CH_3_ group of 5‐CH_3_‐THF is further transferred in several steps to the phenolic OH groups of the phenylpropanoids (Boerjan et al. 2003). However, the exact sources and pathways shaping the isotopic composition of methoxy groups in photosynthetic and heterotrophic plant tissues are not fully resolved.

Previous studies have documented a temporal and spatial strong linear relationship between *δ* ^2^H_meth_ values and the hydrogen isotopic signature of precipitation (*δ* ^2^H_precip_) (Keppler et al. 2007; Anhäuser, Greule, Polag, et al. 2017; Greule et al. 2021). Since *δ* ^2^H_precip_ is primarily influenced by temperature, applying a constant apparent isotope fractionation (ε_app_) between *δ* ^2^H_precip_ and *δ* ^2^H_meth_, makes *δ* ^2^H_meth_ values to a reliable temperature proxy in mid to high latitudes (Wieland et al. 2022; Anhäuser et al. 2020). Greule et al. (2021) reported a mean ε_app_ of −200 ± 14 mUr calculated over 14 different tree species across various European sites, including both angiosperms and gymnosperms. However, Porter et al. (2022) reassessed this data set and suggested different fractionation for angiosperms (−196 ± 14 mUr) and gymnosperms (−204 ± 12 mUr). In addition, Anhäuser, Greule and Keppler (2017) reported fractionation variations for *Picea abies* L., *Pinus sylvestris* L., *Quercus robur* L. and *Fagus sylvatica* L., with *P. abies* showing more than 20 mUr more negative ε_app_ values than the angiosperm species.

Recently, Schuler et al. (2023) found a strong phylogenetic influence on hydrogen isotope fractionation processes of carbohydrates (leaf sugar, *δ* ^2^H_sug_leaf_, and twig cellulose, *δ* ^2^H_cell_twig_) by analysing 73 different tree and shrub species growing in a common garden. They demonstrated that closely related taxa have more similar isotopic signatures and fractionations than distantly related taxa. The results of the studies suggest that *δ* ^2^H values of other plant compounds may also follow phylogenetic patterns. However, investigations comparing the *δ* ^2^H values of carbohydrates and methoxy groups are scarce (Gori et al. 2013; Mischel et al. 2015) and until now, nothing is known about phylogenetic influences on isotope patterns of methoxy groups. Therefore, species‐specific effects should be quantified in methoxy groups for future applicability.

Concerning *δ* ^13^C_meth_, it was observed that *F. sylvatica* and *Larix decidua* Mill. trees reflect summer temperature variations (Riechelmann et al. 2016; Wieland et al. 2022) and are, despite a ^13^C depletion of 4 mUr, documented to be highly correlated with carbon isotopic measurements of cellulose in *Pinus heldreichii* Christ (Wieland et al. 2024). In addition, the study by Gori et al. (2013) compared the hydrogen, oxygen and carbon isotopic signatures of whole wood, cellulose and lignin methoxy groups, using tree‐ring time series (> 70 a) of *P. abies* from three different elevation sites in the south‐eastern Alps (900, 1300 and 1900 m) and showed that the carbon isotopic signatures were highly correlated between the three compounds, while the hydrogen isotopic ratios of methoxy groups correlated to a lesser extent with the hydrogen and oxygen isotopic ratios of whole wood and cellulose. The authors assumed that the plant tissues are influenced by different environmental and biochemical factors. However, until now, the *δ* ^13^C_meth_ values were only measured from a few species and phylogenetic effects could not be considered yet.

Moreover, in the last few decades, several studies found a significant *δ* ^13^C difference in bulk C3 plant material with heterotrophic tissues being ^13^C enriched relative to autotrophic tissues (Craig 1953; Leavitt and Long 1982; Francey et al. 1985; Cernusak et al. 2005, 2009). Several assumptions were considered to explain the ^13^C depletion in leaves (Cernusak et al. 2009) but none of them was able to fully explain the observed differences in several plant compounds. Therefore, further analysis of leaves and heterotrophic material from other compounds such as methoxy groups, may help to further understand the processes leading to the *δ* ^13^C divergences.

However, since most studies analysing the stable isotopes of methoxy groups have focused on tree rings (Gori et al. 2013; Mischel et al. 2015; Riechelmann et al. 2016, 2017; Y. Wang et al. 2020; Wieland et al. 2022), there is limited knowledge about the isotopic composition of leaf methoxy groups. Only one recent study by Cox, Wieland, et al. (2024) published *δ* ^2^H_meth_ and *δ* ^13^C_meth_ values from litter and woody material and documented that the litter was significantly depleted in ^13^C compared to above‐ground wood (twigs and branches with a diameter < 5 mm) and root material. In contrast, *δ* ^2^H_meth_ values showed no significant differences between litter and above‐ground material, but there were significant differences when compared to root material. However, an explicit study of how wood and leaf material within the same plant differ in *δ* ^2^H_meth_ and *δ* ^13^C_meth_ has not been conducted and would provide important information about differences in the lignin synthesis pathway within autotrophic and heterotrophic material.

The aim of this study was to gain a deeper insight into phylogenetic differences of hydrogen and carbon stable isotopes of methoxy groups and to estimate isotopic differences between different plant compounds and tissues to assess insight into metabolic processes of methoxy group synthesis. Therefore, we present *δ* ^2^H_meth_ and *δ* ^13^C_meth_ analyses of woody twig and leaf material of 65 different species growing in a common garden in Basel. As we made use of the plant samples that were already investigated for stable hydrogen measurements of cellulose, sugar, xylem and leaf water (Schuler et al. 2023), we compared the hydrogen signatures of different compounds (twig cellulose, leaf sugar, twig and leaf methoxy groups) and water (xylem and leaf water) across the different plant tissues to assesses isotopic fractionation processes.

In particular, we tested the following hypotheses: (1) Phylogenetic relationships affect the *δ* ^2^H_meth_ and *δ* ^13^C_meth_ values, (2) *δ* ^2^H_meth_ and *δ* ^13^C_meth_ values differ between autotrophic leaf and heterotrophic wood material, (3) Isotope fractionation between tree source water and methoxy groups is similar across phylogenetically different species and (4) Distinct signals are preserved within the stable hydrogen isotope ratios of methoxy groups and carbohydrates as previous proposed by Gori et al. (2013).

## 2 Materials and Methods

### 2.1 Study Site and Sample Material

Leaf and twig samples were collected during the sampling campaign published by Schuler et al. (2023). From this study, we analysed the *δ* ^2^H_meth_ and *δ* ^13^C_meth_ values of leaf and twig material of more than 124 trees and shrubs, 65 species, 18 families and 12 orders (*δ* ^2^H_meth_leaf_, *δ* ^2^H_meth_tiwg_, *δ* ^13^C_meth_leaf_, *δ* ^13^C_meth_twig_). All trees and shrubs were growing in the Kannenfeldpark Basel, Switzerland (47°33054.21600N, 7°34016.12600E), where the small sampling area, the uniform sample conditions and the flat surface led to spatially uniform water access and soil water isotopic signatures. Moreover, the park was regularly irrigated during dry periods, dampening the potential of plant drought stress. Leaf and twig sampling was performed at the end of August 2019 within 2 days. Sun‐exposed twigs were cut from trees with telescopic cutters, put in paper bags, stored on dry ice and dried for 72 h at 60°C. Twig xylem of 2–3‐year‐old twigs was separated from phloem with vegetable peelers and milled to a fine powder using a ball‐mill (Retsch GmbH, Haan, Germany). Whole fully developed leaves were immediately transferred into gas‐tight 12‐mL glass vials (Prod. No. 738W, Exetainer; Labco, Lampeter, UK) and stored on dry ice.

### 2.2 Determination of Stable Methoxy Group Isotopes

The hydrogen and carbon isotope values of leaf and wood material were determined after the method proposed by Greule et al. (2008, 2009). Using this method, the methoxy groups were converted to gaseous iodomethane (CH_3_I) by adding 250 µL of hydriodic acid (HI) to 7 mg of wood and 30 mg of grounded leaf material for *δ* ^2^H_meth_ analysis, and to 5 mg of wood and 20 mg of grounded leaf material for *δ* ^13^C_meth_ analysis. To ensure complete conversion of OCH_3_ to CH_3_I, the samples were heated for 30 min at 130°C in a crimp‐sealed vial, followed by cooling to room temperature.

Isotopic values of *δ* ^2^H_meth_ and *δ* ^13^C_meth_ were determined by injecting 10–90 µL (gas‐tight syringe, Trajan, 100 µL) of the headspace gas using an autosampler (A200S, CTC Analytics, Zwingen, Switzerland) into a gas chromatography thermal conversion or combustion isotope mass spectrometry (GC‐TC‐IRMS, GC‐C‐IRMS), respectively.

The gas chromatographs (HP6890N, Agilent, Santa Clara, USA, for *δ* ^2^H_meth_, and Trace GC, Thermo Finnigan, Milan, Italy, for *δ* ^13^C_meth_ measurement) were each equipped with a DB‐5MS Agilent J&W capillary column (length: 30 m, internal diameter: 0.25 mm, film thickness: 0.5 μm). For *δ* ^2^H_meth_ analysis, a 4:1 split injection was used with helium as the carrier gas at a constant flow rate of 0.7 mL min⁻¹. CH_3_I was converted into molecular hydrogen (H_2_) in a thermo conversion reactor (Al_2_O_3_, 320 mm length, 0.5 mm internal diameter) at 1450°C. For *δ* ^13^C_meth_ analysis, a 15:1 split injection was used, with helium as the carrier gas at a constant flow rate of 1.8 mL min⁻¹. CH_3_I was oxidised to CO_2_ at 960°C in an oxidation reactor (Al_2_O_3_, 320 mm length, 0.5 mm internal diameter, containing Cu, Ni and Pt wires). The resulting H_2_ or CO_2_ gases were transferred through a GC–Combustion III interface (ThermoQuest Finnigan) into the IRMS (DeltaplusXL, ThermoQuest Finnigan). High‐purity hydrogen gas (Alphagaz 2 H_2_, Air Liquide, Düsseldorf, Germany) was used as the monitoring gas, and the H_3_^+^ factor was measured daily, remaining below 3 ppm/nA throughout the measurement period. For *δ* ^2^H_meth_ analysis, a correction function was applied to account for area‐dependent variations. To model this effect, varying amounts of CH_3_I, spanning the range of sample peak areas, were measured before and after each sequence and used to perform a linear regression.

The isotope values were normalised by a two‐point linear calibration using two reference materials covering the whole range of the measured isotopic values. The specific characteristics of the reference material were as follows: for *δ* ^2^H_meth_ measurements, sodium methyl sulphate HUBG1 (−144.5 ± 1.2 mUr) and birch wood material HUBG3 (−272.9 ± 1.5 mUr), and for *δ* ^13^C_meth_ measurements, potassium methyl sulphate HUBG2 (+1.60 ± 0.12 mUr) and beech wood material HUBG4 (−30.17 ± 0.13 mUr). The reference material was previously measured by IRMS analyses (IsoLab, Max Planck Institute of Biogeochemistry) and calibrated to the VSMOW scale for *δ* ^2^H_meth_ and to the Vienna Pee Dee Belemnite (VPDB) scale for *δ* ^13^C_meth_ values (Greule et al. 2019, 2020). Both reference materials were consecutively injected after every sixth plant sample, respectively. The precision of the measurement system for *δ* ^13^C and *δ* ^2^H measurements was ±0.3 and ±3 mUr, respectively.

### 2.3 Additional Isotope Data Collection Related to the Common Garden Experiment

For enabling a tree‐specific comparison between the isotopic composition of xylem water (*δ* ^2^H_xw_), carbohydrates and methoxy groups, we used the *δ* ^2^H_xw_, *δ* ^2^H_sug_leaf_ and *δ* ^2^H_cell_twig_ data from Schuler et al. (2023), which were derived from the exact same trees. Please note that the study of Schuler et al. (2023) exclusively analysed twig material from the year 2019. *δ* ^2^H_xw_ values were measured using the cryogenic vacuum distillation (CVD). Recently, some studies observed that *δ* ^2^H_xw_ values obtained by CVD are significantly lower than the hydrogen isotope composition of the plant source water. This offset is less driven by fractionation processes during plant water uptake than by cryogenic extraction artefacts that bias the determination of *δ* ^2^H_xw_ values (Allen and Kirchner 2022). To avoid large enrichment biases by CVD, *δ* ^2^H_xw_ values were measured after the method of West et al. (2006) but adapted after the suggestions of Diao et al. (2022). Potential bias of the CVD method should therefore be negligible. For more information on the materials and methods used for the stable isotope measurements of carbohydrates and xylem water, we refer the reader to the study of Schuler et al. (2023).

### 2.4 Quantification of Lignin Methoxy Groups in Leaf Material

While the major fraction of wood OCH_3_ originates from lignin (ether bounded), the polysaccharide pectin can also be a major source of OCH_3_ in other plant tissues (ester bounded). Leaves contain a much higher amount of pectin than wood (between 7% and 35% of the cell wall material, Keppler et al. [2004]) and measurements of bulk methoxy groups from leaves reflect both the lignin and pectin OCH_3_ pool. To consider only lignin *δ* ^2^H_meth_leaf_ values, alkaline hydrolysis can be used to selectively remove the ester‐bounded (pectin) OCH_3_, enabling the analysis of the residual ether‐bounded OCH_3_ (Greule and Keppler 2011; Cox, Laceby, et al. 2024; Cox, Wieland, et al. 2024). For this, 500 µL NaOH (2 molar) was added to 30 mg leaf material in a 1.5 mL crimp cap vial. The vials were heated at 60°C for 12 h and subsequently opened and dried for at least 24 h. The generated gaseous methanol (CH_3_OH) is released to the ambient air, and only ether‐bound methoxy groups are left. Neutralisation was done by adding fuming HCl for more than 24 h. For the subsequent lignin *δ* ^2^H_meth_leaf_ measurement, the residue material was treated and measured as described in Section (2.2).

### 2.5 Data Treatment and Statistics

The delta (*δ*) notation is used for all hydrogen and carbon isotope values. In this study, we used the unit ‘Urey’ for expressing isotope values (Ur, Urey [1948], suggested by Brand and Coplen [2012]) (1 mUr is equivalent to 1‰). *δ* ^2^H values were calculated as the deviation from the Vienna Standard Mean Ocean Water (VSMOW) and *δ* ^13^C values from the VPDB.

All statistical analyses were performed using R v4.4.1 (R Core Team 2024). The impact of phylogeny on methoxy groups was λ an estimation to which degree the evolutionary history of the species effects the isotopic signature. Pagel's λ range between 0 and 1, with higher values indicating a stronger influence of phylogeny. The phylogenetic trees were generated according to the template provided by Schuler et al. (2023) utilising the R package PHYTOOLS. Due to the uneven number of replicates per species, the mean values were used. Analyse of variances (ANOVA) between species, orders, families and genera followed the Tukey's post hoc test. The Tukey test as well as the linear models to determine the coherences between the different plant components were implemented in the R package stats.

Apparent isotope fractionation between *δ* ^2^H_meth_twig_ and tree source water (*δ* ^2^H_xw_ or *δ* ^2^H_precip_) was calculated as given in Equation (1) with isotope values given in [Ur]:

(1) $${Ɛ}_{\text{app}}=(\delta^{2}H_{\text{meth\_twig}}\;[\text{Ur}]+1)/{(\delta }^{2}H_{\text{xw}/\text{precip}}[\text{Ur}]+1)-1$$

*δ* ^2^H_precip_ values were obtained from PISO.AI, a machine learning model that uses geographical and climate data to provide monthly time series of precipitation isotope values for any location in Europe (Nelson et al. 2021).

## 3 Results

### 3.1 *δ* ^2^H and *δ* ^13^C Values of Leaf and Twig Methoxy Groups

**Figure 1:**
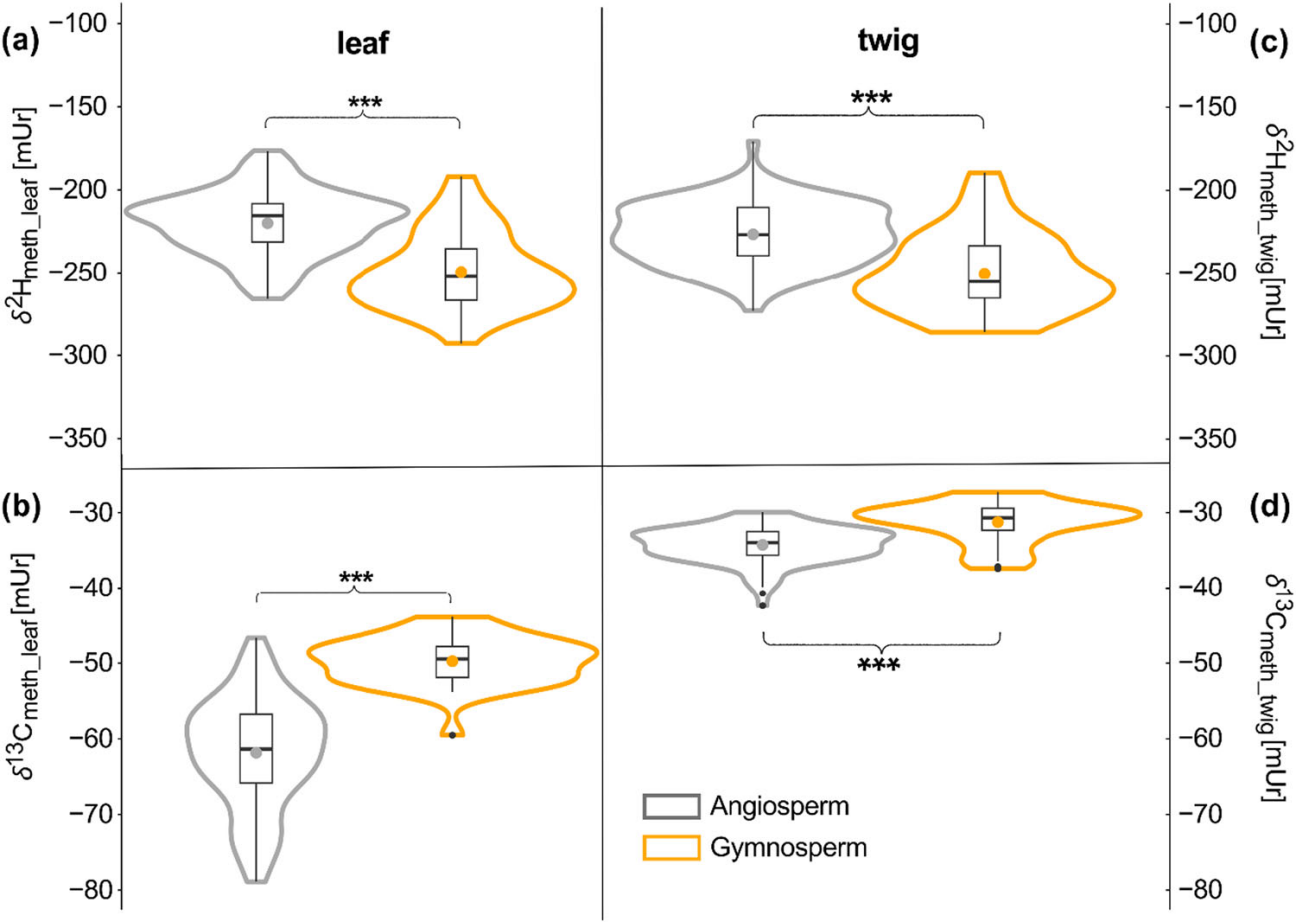
Violine plots of hydrogen (*δ* ^2^H_meth_,) (a, c) and carbon (*δ* ^13^C_meth_) (b, d) isotope ratios of leaf (a, b) and twig (c, d) methoxy groups across individuals of 65 trees and shrubs species. In all panels angiosperms (grey) and gymnosperm (orange) are significantly different (*p* < 0.001, indicated with asterisks). The boxplots within the violin plots are showing the mean (points) and median (horizontal line) values with whiskers representing the 95% confidence interval (CI). [Color figure can be viewed at wileyonlinelibrary.com]

The *δ* ^2^H_meth_ and *δ* ^13^C_meth_ values from leaf and twig material of the individual trees were normally distributed, with predominantly unimodal peaks around the mean and the mean being close to the median (Figure 1). Mean *δ* ^2^H_meth_ values in leaves and twigs were −221.5 ± 17.8 and −226.2 ± 17.3 mUr for angiosperms, while values for gymnosperms for both tissues were significantly different and averaged around −247 ± 22 mUr (Table 1, Figure 1a,c). In contrast, mean *δ* ^13^C_meth_ values differed strongly between leaves and twigs with −62.4 ± 6.7 and −34.2 ± 2 mUr in angiosperms compared to −49.8 ± 3.5 and −31.2 ± 2.7 mUr in gymnosperms. While variances within *δ* ^2^H_meth_, *δ* ^13^C_meth_twig_ and *δ* ^13^C_meth_leaf_ gymnosperm were similar, angiosperm *δ* ^13^C_meth_leaf_ values ranged more widely from −78.9 to −46.8 mUr, showing a slightly bimodal distribution with a secondary accumulation around −72 mUr (Figure 1b).

**Table 1:** Overall species and individual seed type means of hydrogen (*δ* ^2^H_meth_) and carbon (*δ* ^13^C_meth_) isotope values of leaf and twig methoxy groups. Δ represents the offset between leaf and twig methoxy groups and ε the isotope fractionation between xylem water and methoxy groups (ε_meth_xw_), xylem water and cellulose (ε_cell_xw_) and precipitation and methoxy groups (ε_meth_precip_). Pagel's λ illustrates the strength of the phylogenetic effect with bold numbers representing highly significant values (*p* < 0.05).

	Mean values									Pagel's λ							
	*δ* ^2^H_meth_		*δ* ^13^C_meth_		ΔH_leaf‐twig_	ΔC_leaf‐twig_	ε_meth_xw_	ε_meth_precip_	ε_cell_xw_	*δ* ^2^H_meth_		*δ* ^13^C_meth_		ΔH_leaf‐twig_	ΔC_leaf‐twig_	ε_meth_xw_	ε_meth_precip_
	Leaf	Twig	Leaf	Twig						Leaf	Twig	Leaf	Twig				
Species mean	−229	−233	−59.2	−33.2	3.4	−25.6	−192	−184	5.5	**0.73**	**0.62**	**0.79**	**0.47**	**0.65** a	**0.79**	**0.64**	**0.62**
Angiosperm	−222	−226	−62.4	−34.2	6.2	−27.8	−184	−193	10.8	**0.79**	< 0.01	**0.79**	0.24	0.64	**0.76**	< 0.01	< 0.01
Gymnosperm	−247	−247	−49.8	−31.2	0.3	−19.1	−209	−199	−4.9	< 0.01	0.31	< 0.01	0.48	< 0.01	**0.99**	0.31	0.31

*Note:* Bold numbers representing highly significant values *p* < 0.05.

^a^ Excluding *Magnolia kobus*.

### 3.2 Phylogenetic Variations Across Stable Methoxy Isotope Ratios

**Figure 2:**
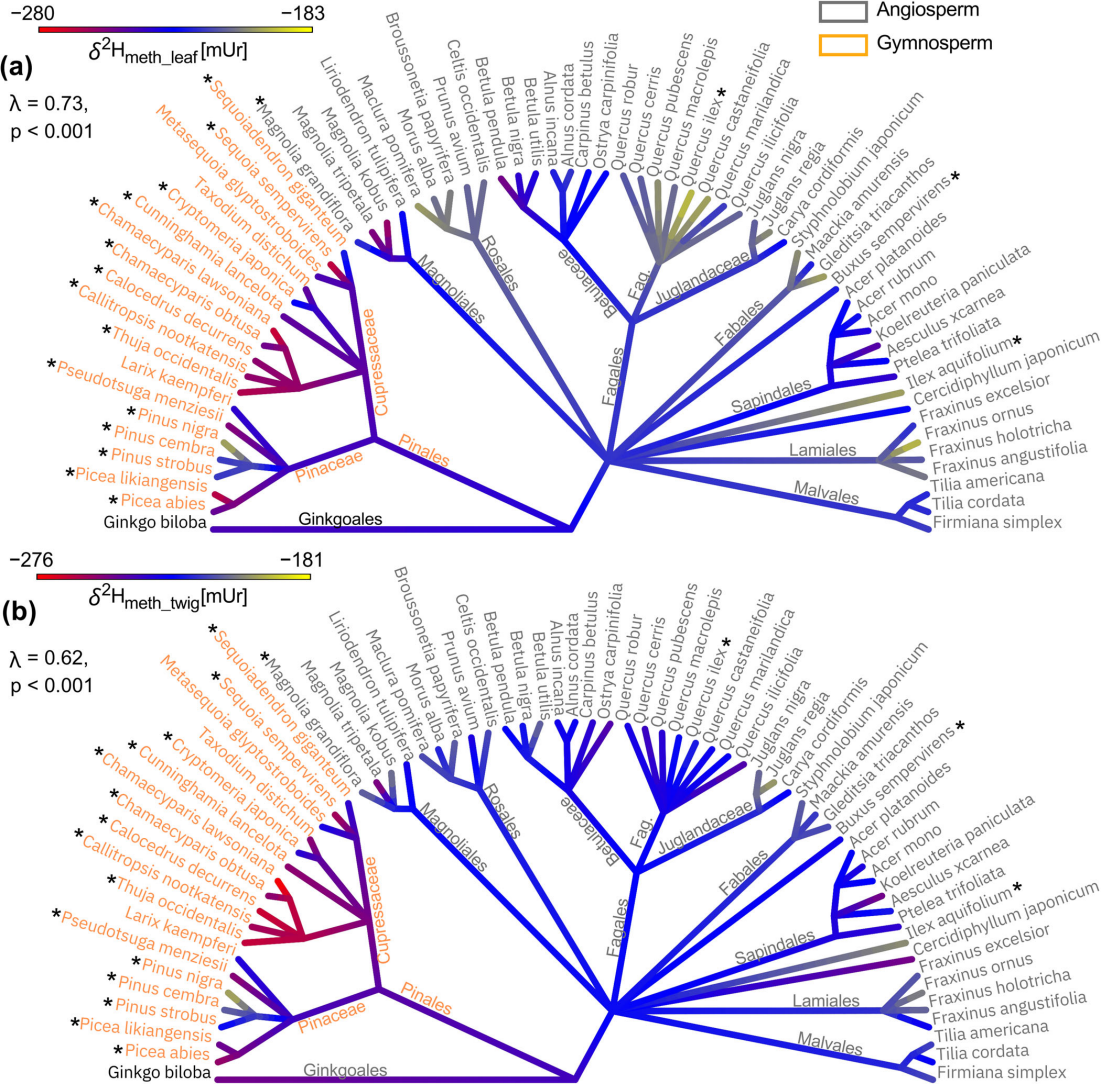
Phylogenetic trees showing hydrogen isotope ratios of methoxy groups from (a) leaves (*δ* ^2^H_meth_leaf_) and (b) twigs (*δ* ^2^H_meth_twig_). Gymnosperms are coloured in orange and angiosperms in grey, asterisks indicate evergreen species. λ shows Pagel's λ used to estimate the phylogenetic signal, with corresponding *p* value for significance estimation. [Color figure can be viewed at wileyonlinelibrary.com]

The phylogenetic effect of *δ* ^2^H_meth_ differed between the two plant tissues, with *δ* ^2^H_meth_leaf_ showing a higher Pagel's λ of 0.73 (*p* < 0.001) compared to *δ* ^2^H_meth_twig_ λ = 0.62 (*p* < 0.001) (Table 1, Figure 2, Figure S1). The highest *δ* ^2^H_meth_ values were recorded in *Ilex aquifolium* L. in leaves (−181 mUr) and twigs (−183 mUr), while the lowest values were observed in *Sequoia sempervirens* (D.Don) Endl. leaves (−280 mUr) and in *Chamaecyparis lawsoniana* A. Murr. Parl. twigs (−276 mUr). The phylogenetic trees and the corresponding ANOVA results revealed distinct patterns among the tested phylogenetic groups, particularly between angiosperms and gymnosperms. In *δ* ^2^H_meth_leaf_, the orders Rosales, Fagales, Lamiales and Aquifoliales were significantly ^2^H enriched compared to Pinales, while the *δ* ^2^H_meth_leaf_ values for Ginkgoales, Magnoliasles, Fabales, Buxales and Sapindales ranged between them (Figure 2a, Table 2). In *δ* ^2^H_meth_twig_ the phylogenetic groups showed more homogeneity (Figure 2b), with only Pinales and Saxifragales being significantly more depleted in ^2^H, and Aquifoliales being significantly enriched in ^2^H compared to other angiosperm orders (Table 2). However, it is important to note, that the orders Aquifoliales and Saxifragales are represented by a single species with only two and one data points, respectively. Ginkgoales, though neither an angiosperm nor a gymnosperm, showed *δ* ^2^H_meth_ values more similar to angiosperm species.

**Table 2:** Order level mean hydrogen (*δ* ^2^H_meth_) and carbon (*δ* ^13^C_meth_) values of leaf and twig methoxy groups, offsets between leaf and twig methoxy groups (Δ), compact letter display used to illustrate significant differences post‐ANOVA (significance threshold = 0.05) and ^2^H fractionations between xylem water and methoxy groups (ε_meth_xw_), and xylem water and cellulose (ε_cell_xw_).

Orders	Mean values						Compact letter display						ε_meth/xw_	ε_cell/xw_
	*δ* ^2^H_meth_		*δ* ^13^C_meth_		ΔH_leaf‐twig_	ΔC_leaf‐twig_	*δ* ^2^H_meth_		*δ* ^13^C_meth_		ΔH_leaf‐twig_	ΔC_leaf‐twig_		
	Leaf	Twig	Leaf	Twig			Leaf	Twig	Leaf	Twig				
Ginkgoales	−253	−260	−59.4	−31.1	6.2	−28.3	ab	ab	abcd	ab	a	abcd	−217	−1
Pinales	−246	−247	−49.8	−31.2	0.9	−18.6	b	b	a	a	a	a	−209	−5
Magnoliales	−242	−226	−54.4	−33.5	−15	−21	ab	ab	ab	ab	a	ab	−185	11
Rosales	−208	−220	−64.4	−34.6	11.6	−29.8	a	ab	bcd	ab	a	bcd	−176	11
Fagales	−220	−230	−59.1	−33.3	9.7	−25.8	a	ab	b	ab	a	bc	−188	5
Fabales	−210	−214	−70.5	−35.0	4.1	−35.5	ab	ab	cd	ab	a	d	−168	12
Buxales	−230	−237	−54.3	−35.5	6.6	−18.8	ab	ab	abc	ab	a	abc	−200	0
Sapindales	−237	−235	−66.3	−36.0	−2.6	−30.4	ab	ab	cd	b	a	cd	−190	13
Aquifoliales	−183	−181	−68.3	−33.7	−2.0	−34.6	a	a	bcd	ab	a	bcd	−139	25
Saxifragales	−238	−272	−60.3	−32.9	34.4	−27.4	ab	b	abcd	ab	a	abcd	−236	25
Lamiales	−211	−215	−73.5	−34.9	4.2	−38.6	a	ab	d	ab	a	d	−173	34
Malvales	−222	−220	−62.0	−35.1	−1.9	−26.9	ab	ab	bcd	ab	a	bcd	−177	6

**Figure 3:**
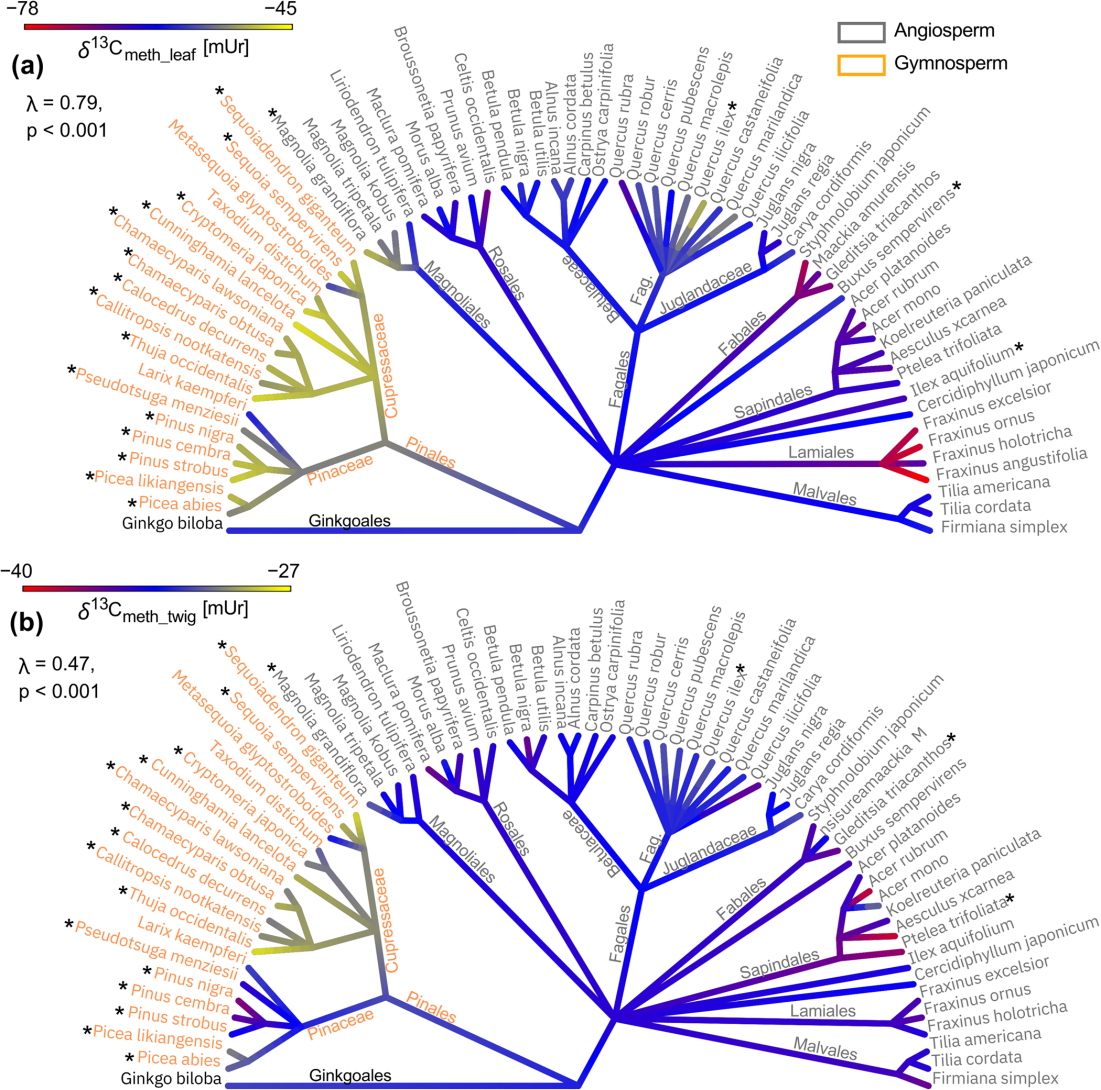
Phylogenetic trees showing carbon isotopes of methoxy groups from (a) leaves (*δ* ^13^C_meth_leaf_) and (b) twigs (*δ* ^13^C_meth_twig_). Gymnosperms are coloured in orange and angiosperms in grey, asterisks indicate evergreen species. λ shows Pagel's λ used to estimate the phylogenetic signal, with corresponding *p* value for significance estimation. [Color figure can be viewed at wileyonlinelibrary.com]

The phylogenetic effect on *δ* ^13^C_meth_ values showed a different and more complex pattern, particularly in the leaf material (λ = 0.79, *p* < 0.001) (Figure 3). *Fraxinus angustifolia* Vahl recorded the most negative *δ* ^13^C_meth_ values in leaves (−78.3 mUr) and *Aesculus xcarnea* Hayne in twigs (−39.6 mUr), while *Cunninghamia lancelota* (Lamb.) Hook. leaves (−44.8 mUr) and *Thuja occidentalis* L. twigs (−27.2 mUr) had the least negative values.

Significant differences were noted between angiosperms and gymnosperms, with Pinales being notably ^13^C enriched compared to most angiosperm orders. Within angiosperms, orders like Lamiales, Fabales and Sapindales were significantly depleted in ^13^C. Deciduous gymnosperms such as *Larix kaempferi* (Lamb.) Carrière and *Metasequoia glyptostroboides* Hu & Cheng had *δ* ^13^C_meth_leaf_ values similar to angiosperms. Evergreen angiosperms like *Quercus ilex* L. and *Magnolia grandiflora* L. were more ^13^C depleted than the average angiosperm species, while the evergreen *I. aquifolium* had values close to the mean angiosperm species. In *δ* ^13^C_meth_twig_ values (Figure 3b) a lower λ value was estimated (0.47, *p* < 0.001) and significant differences were only observed between the Pinales and Sapindales order (*p* < 0.05, Table 2).

### 3.3 Comparison of Hydrogen and Carbon Isotope Ratios Between Leaf and Twig Methoxy Groups

**Figure 4:**
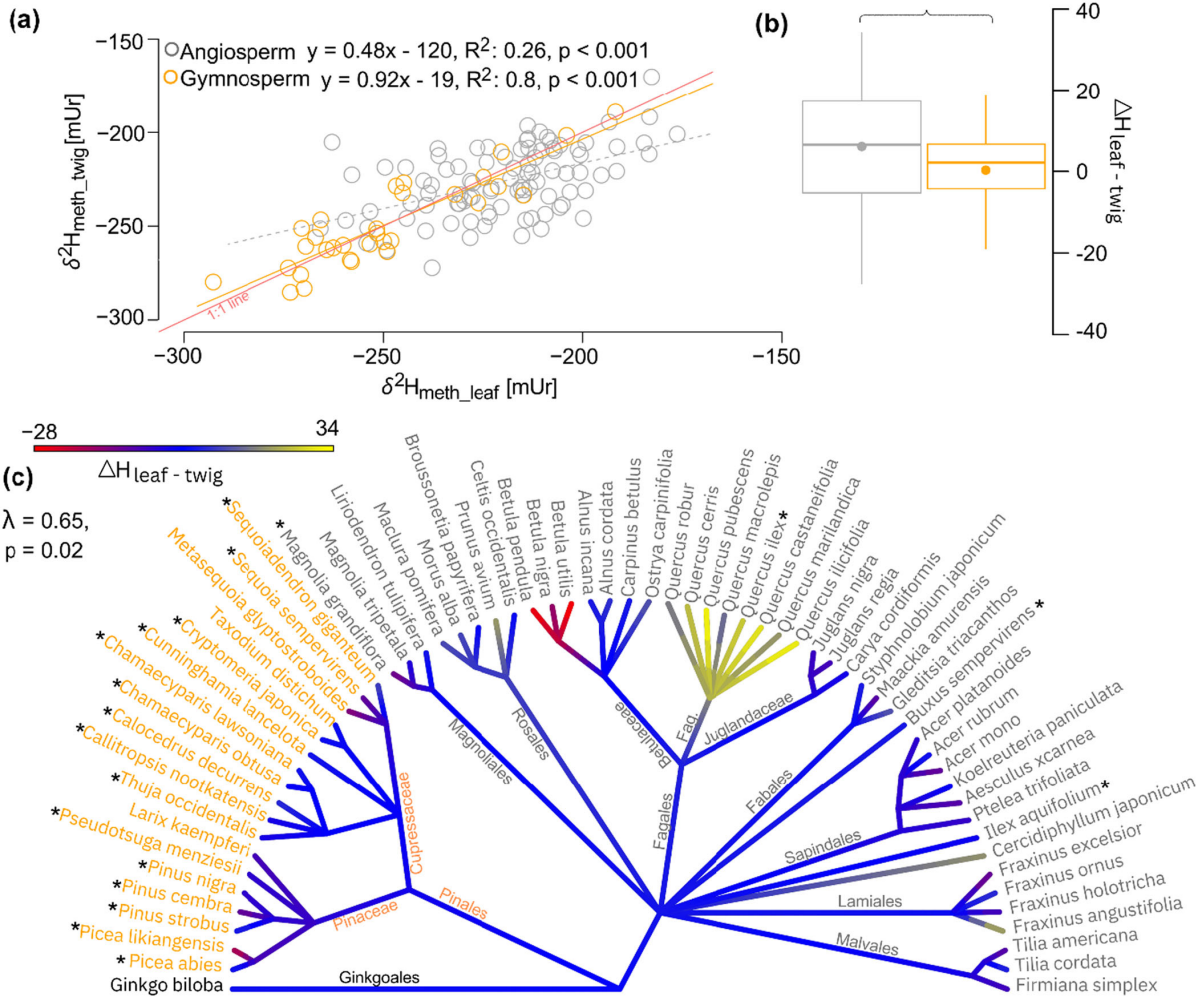
(a) Linear correlation between hydrogen isotopes of methoxy groups in leaves (*δ* ^2^H_meth_leaf_) and twigs (*δ* ^2^H_meth_twig_). The red solid line represents the 1:1 ratio, while the grey dotted line shows the linear correlation for angiosperms, and the orange solid line represents the linear correlation for gymnosperms. (b) Boxplots illustrating the difference between leaf and twig methoxy groups (ΔH_leaf‐twig_), with points indicating the mean values and horizontal lines indicating the medians. (c) Phylogenetic tree displaying ΔH_leaf‐twig_ values, with asterisks marking evergreen species. The symbol λ represents Pagel's λ, used to estimate the phylogenetic signal, along with the corresponding *p* value for significance. In all plots, gymnosperms are shown in orange and angiosperms in grey. [Color figure can be viewed at wileyonlinelibrary.com]

*δ* ^2^H_meth_ values of leaves and twigs were highly significantly correlated (*r* = 0.51, *p* < 0.001), whereby gymnosperms correlated to a much higher extent than angiosperms (Figure 4a). In gymnosperm species 80% of the variations within *δ* ^2^H_meth_leaf_ values can be explained by *δ* ^2^H_meth_twig_ values (*p* < 0.001), closely following a 1:1 line. The covariance within angiosperms was significantly lower, where *δ* ^2^H_meth_leaf_ only explain 26% of the variations in *δ* ^2^H_meth_twig_ values.

*δ* ^2^H differences between leaves and twigs (ΔΗ_leaf‐twig_) ranged around 0 with angiosperms: 6.2 ± 15.8 mUr and gymnosperms: 0.32 ± 9.9 mUr (Table 1). ΔΗ_leaf‐twig_ variances within the seed types were much greater in angiosperms than in gymnosperm, with angiosperms ranged from −57.5 (*Magnolia kobus* DC. not shown in Figure 4b due to its extreme negative value) to +34.3 mUr (*Cercidiphyllum japonicum* Siebold & Zucc. Ex J.J.Hoffm. & J.H.Schult.Bis and gymnosperms from −19.1 (*Picea likiangensis* [Franch.] E. Pritz) to +18.7 mUr (*Sequoiadendron giganteum* J.Buchholz) (Figure 4b).

ΔΗ_leaf‐twig_ values showed a significant phylogenetic λ of 0.65 (*p* = 0.02) but no significant variations between the different orders (Figure 4c, Table 2). However, on family level, the angiosperm Fagacea family showed strong differences compared to the other angiosperm species with ΔΗ_leaf‐twig_ values up to +34 mUr.

**Figure 5:**
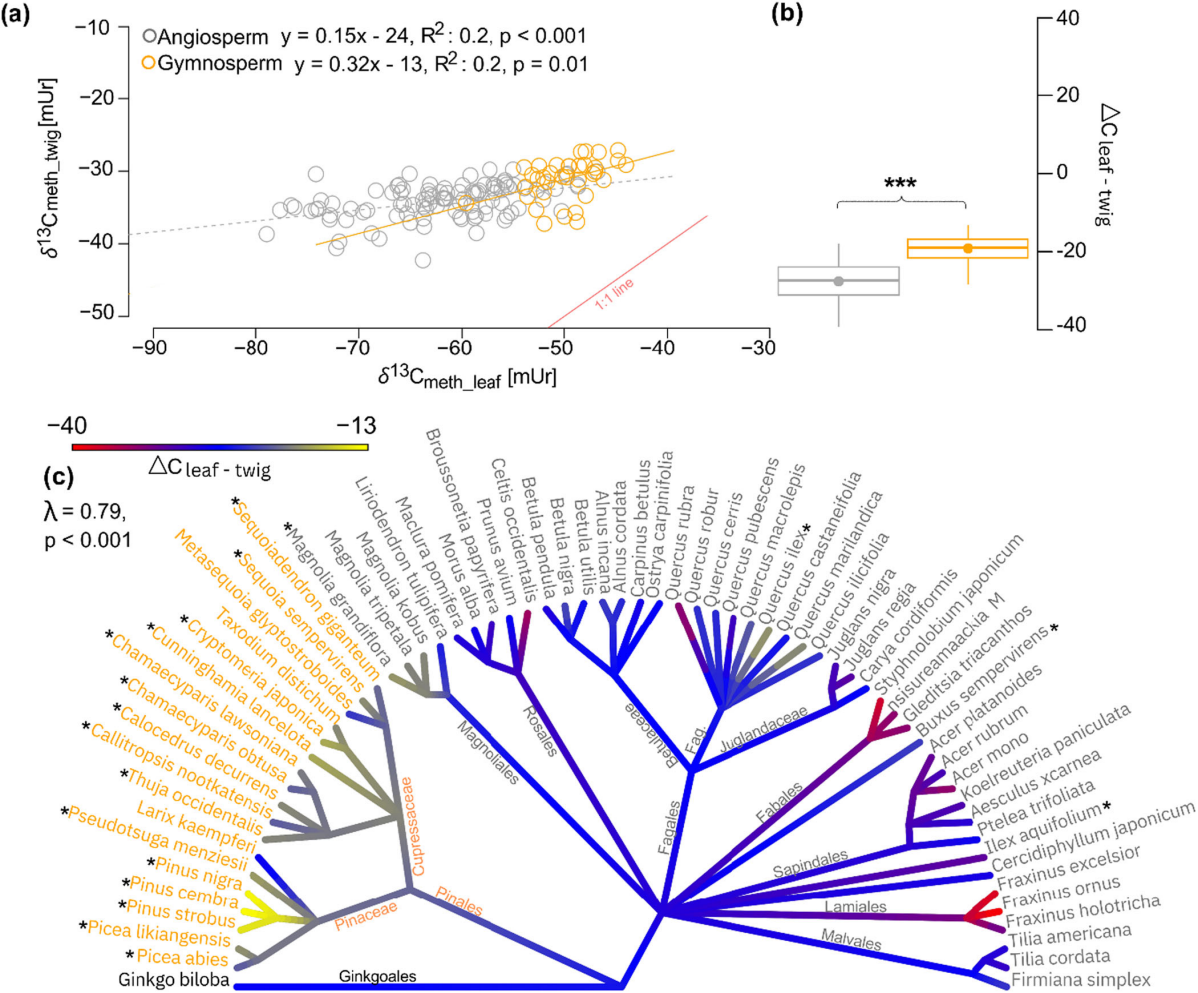
(a) Linear correlation between carbon isotope ratios of methoxy groups in leaves (*δ* ^13^C_meth_leaf_) and twigs (*δ* ^13^C_meth_twig_). The red solid line represents the 1:1 ratio, while the grey dotted line shows the linear correlation for angiosperms, and the orange solid line for gymnosperms. (b) Boxplots illustrating the difference between leaves and twigs methoxy groups (ΔC_leaf‐twig_), with points representing the mean values and horizontal lines indicating the medians. Asterisks denote the significance level between angiosperms (grey) and gymnosperms (orange), with *** indicating *p* < 0.001. (c) Phylogenetic tree displaying ΔC_leaf‐twig_ values, with asterisks marking evergreen species. The symbol λ represents Pagel's λ, used to estimate the phylogenetic signal, along with the corresponding *p* value for significance. In all plots, gymnosperms are shown in orange and angiosperms in grey. [Color figure can be viewed at wileyonlinelibrary.com]

The *δ* ^13^C_meth_ values of leaf and twig material showed low but significant linear relationships for angiosperms (*R* ^2^ = 0.2, *p* < 0.001) and for gymnosperms (*R* ^2^ = 0.2, *p* = 0.01) (Figure 5a). The *δ* ^13^C_meth_leaf_ material was significantly lower than *δ* ^13^C_meth_twig_, with a mean ΔC_leaf‐twig_ value for angiosperm = −27.8 ± 5.8 mUr and for gymnosperm = −19.1 ± 3.72 mUr (Figure 5b, Table 1).

In ΔC_leaf‐twig_ a highly significant λ = 0.79, *p* < 0.001 (Figure 5c) was documented with ΔC_leaf‐twig_ in gymnosperms being lower than in angiosperms. For angiosperms the orders Fabales and Lamiales had the most negative ΔC_leaf‐twig_ values, while Magnoliales showed the least negative values. For gymnosperms *Pinus nigra* J.F. Arnold, *Pinus cembra* L. and *Pinus strobus* L. showed much less negative ΔC_leaf‐twig_ values than the other species belonging to the Pinaceae or Cupressaceae family (Figure 5).

**Figure 6:**
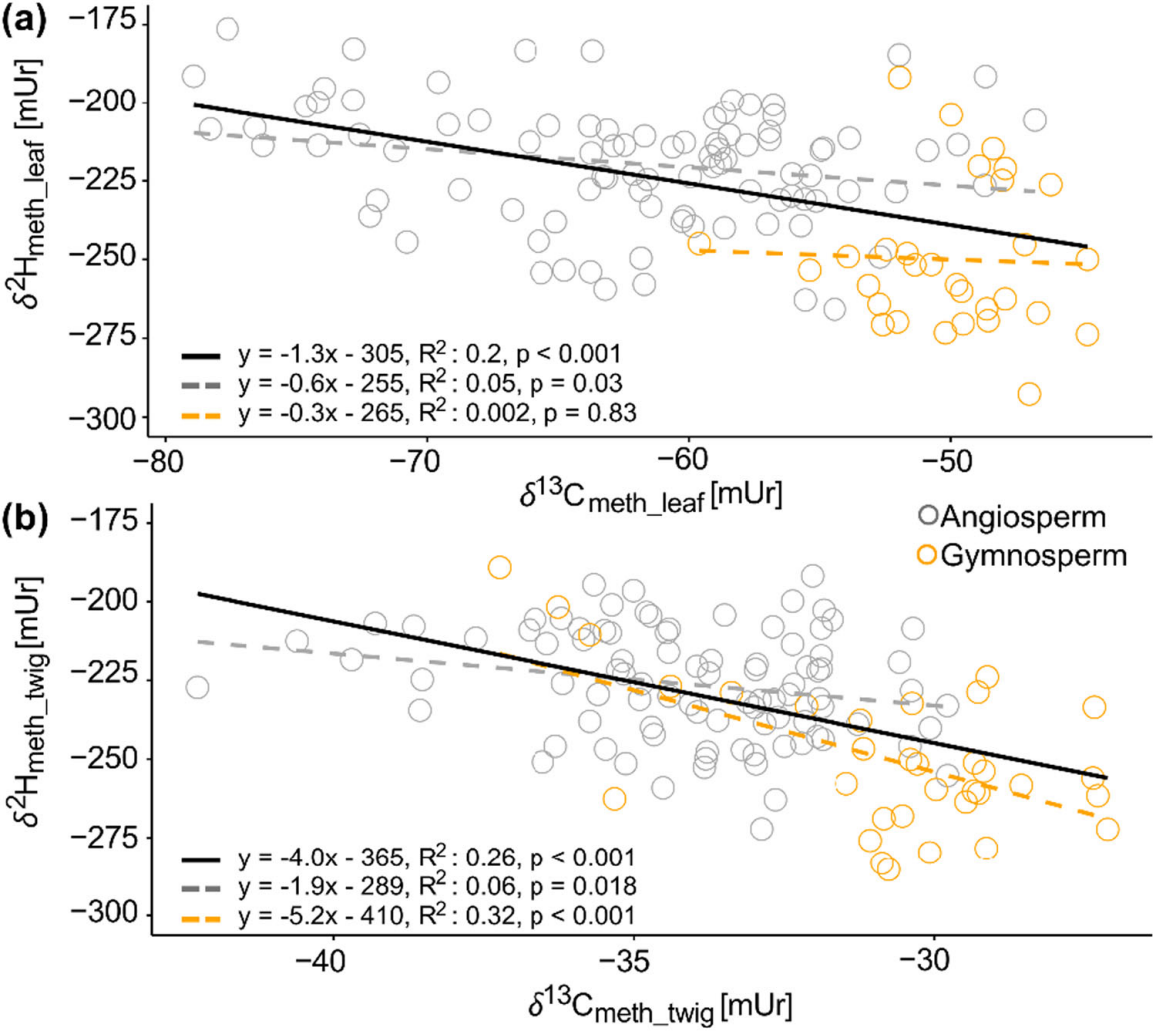
Linear correlations between carbon and hydrogen isotope ratios of methoxy groups from (a) leaves (*δ* ^13^C_meth_leaf_; *δ* ^2^H_meth_leaf_) and (b) twigs (*δ* ^13^C_meth_twig_; *δ* ^2^H_meth_twig_). In both plots, gymnosperms are coloured in orange and angiosperms in grey. Black solid lines represent the linear correlation between *δ* ^2^H_meth_ and *δ* ^13^C_meth_ values, and dashed lines separate between angiosperm (grey) and gymnosperm (orange) species. [Color figure can be viewed at wileyonlinelibrary.com]

Comparison between *δ* ^2^H_meth_ and *δ* ^13^C_meth_ values showed a significant negative correlation within both leaf and twig methoxy groups (Figure 6), whereas angiosperm leaf and twig material cover a much wider range than gymnosperms for both carbon and hydrogen isotope ratios. However, coefficient of determination became lower and non‐significant when separating angiosperms and gymnosperms (except for gymnosperm twigs).

### 3.4 Hydrogen Isotopes of Methoxy Groups and Carbohydrates

**Figure 7:**
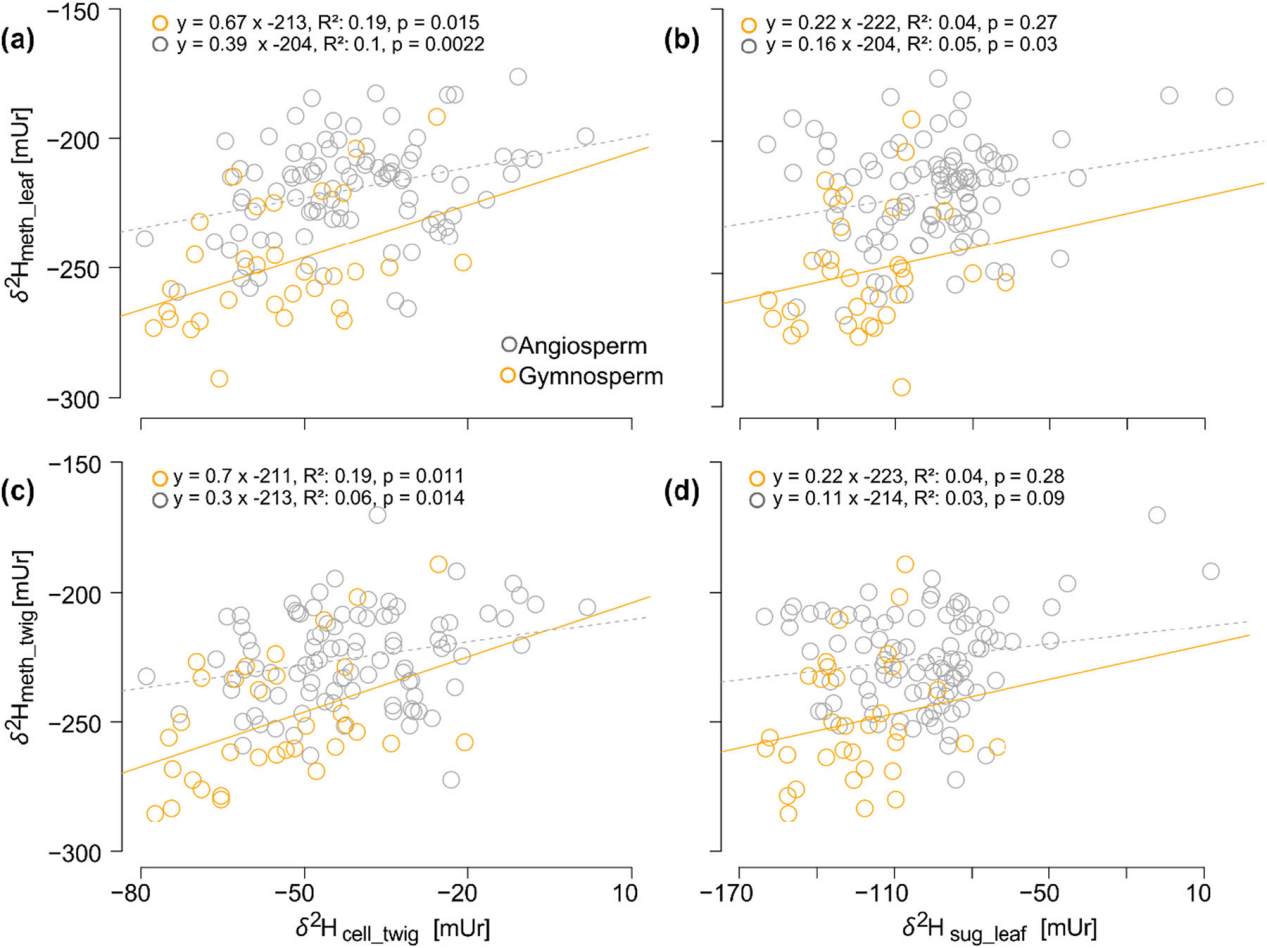
Linear relationship between hydrogen isotope values of (a) twig cellulose (*δ* ^2^H_cell_twig_) and leaf methoxy groups (*δ* ^2^H_meth_leaf_), (b) leaf sugar (*δ* ^2^H_sug_leaf_) and leaf methoxy groups, (c) twig cellulose and twig methoxy groups (*δ* ^2^H_meth_twig_) and (d) leaf sugar and twig methoxy groups. In all plots, gymnosperms are coloured in orange and angiosperms in grey. The orange solid line represents the linear correlation between gymnosperms and the dashed grey line the linear correlation between angiosperms. [Color figure can be viewed at wileyonlinelibrary.com]

In this section, we compare the *δ* ^2^H_meth_ values of this study with those of *δ* ^2^H_sug_leaf_, *δ* ^2^H_cell_twig_ and *δ* ^2^H_xw_ values from the same trees previously published by Schuler et al. (2023). *δ* ^2^H_meth_twig_ values were in mean −187.5 mUr lower than *δ* ^2^H_cell_twig_ values. While *δ* ^2^H_meth_twig_ ranged from −276.4 to −181 mUr, *δ* ^2^H_cell_twig_ ranged around −45.2 ± 14.8 mUr. *δ* ^2^H_meth_leaf_ values were in mean −121.9 mUr lower than *δ* ^2^H_sug_leaf_ values and values ranged from −279.9 to −183 mUr in *δ* ^2^H_meth_leaf_ and −160.8 to −15.1 mUr in *δ* ^2^H_sug_leaf_. Correlations between *δ* ^2^H values of carbohydrates and methoxy groups are low in both twig and leaf material with highest correlations between angiosperm *δ* ^2^H_meth_leaf_ and *δ* ^2^H_cell_twig_ (*R* ^2^ = 0.19, *p* = 0.015) and gymnosperm *δ* ^2^H_meth_twig_ and *δ* ^2^H_cell_twig_ values (*R* ^2^ = 0.19, *p* < 0.011) (Figure 7).

**Figure 8:**
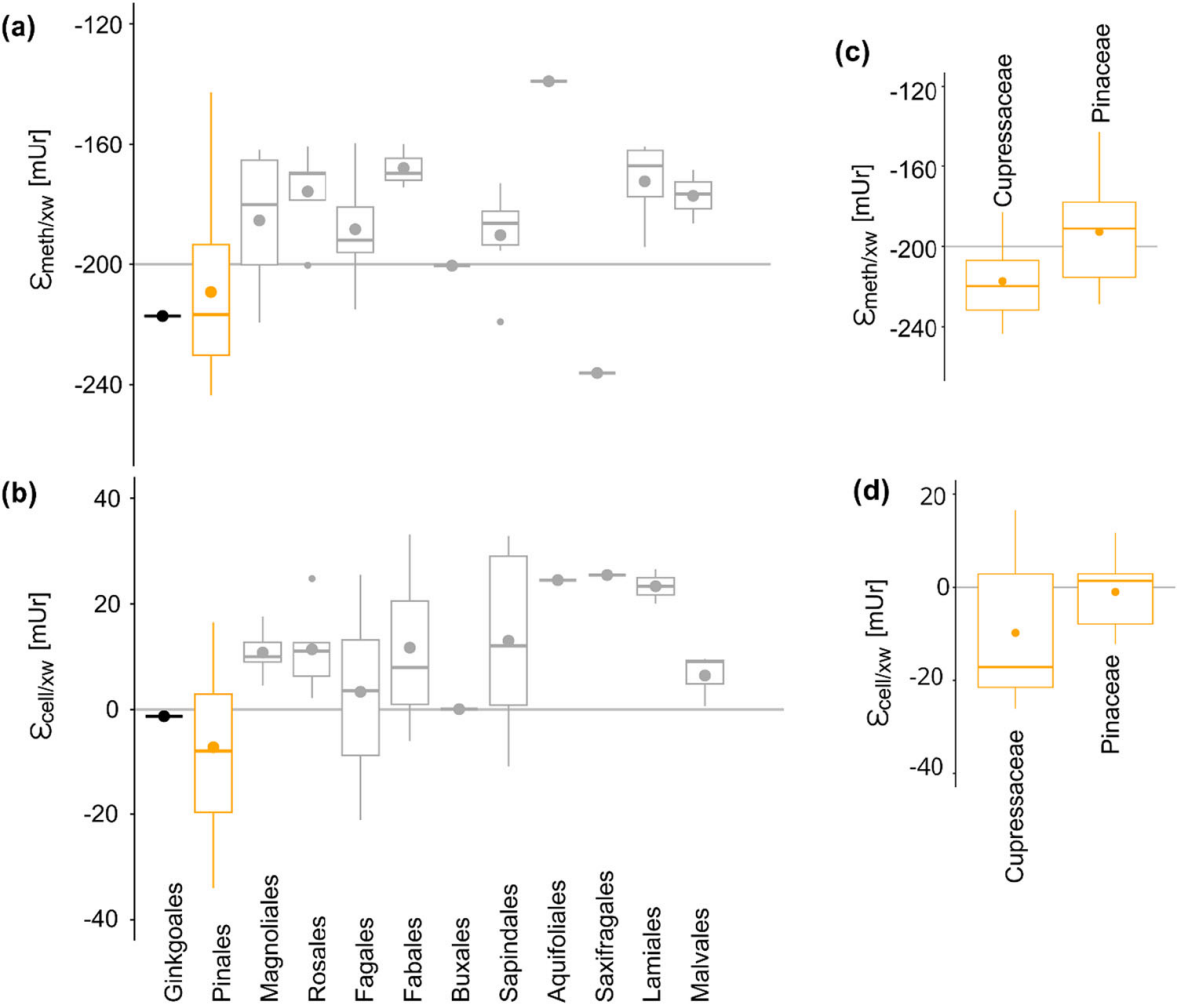
Order level fractionation between hydrogen isotope values of (a) twig methoxy groups and xylem water (ε_meth/xw_) with the grey solid line representing the documented apparent fractionation of −200 mUr by Greule et al. (2021). (b) Fractionation between twig cellulose and xylem water (ε_cell/xw_) with the grey solid line representing no isotopic fractionation (0). Family level fractionation of Pinales (c) ε_meth/xw_ and (d) ε_cell/xw_. In all plots gymnosperms are coloured orange and angiosperm in grey and the boxplots showing the mean (points) and median (horizontal line) values, whiskers representing the 95% CI. [Color figure can be viewed at wileyonlinelibrary.com]

Isotope fractionations were calculated between *δ* ^2^H_meth_twig_ and *δ* ^2^H_xw_ (ε_meth/xw_) (Figure 8a) and compared with isotopic fractionation between *δ* ^2^H_cell_twig_ and *δ* ^2^H_xw_ (ε_cell/xw_) (Table 2, Figure 8b). The mean ε_meth/xw_ value for angiosperm was −184 ± 19 mUr, while gymnosperms showed a more depleted mean of −209 ± 26 mUr (Table 1). Similar to previous observations of *δ* ^2^H_meth_twig_ values (Figure 2), higher species differences were documented in the gymnosperms than in the angiosperms. Within the Pinales, the ε_meth/xw_ values ranged from −143 to −232 mUr, with the Cupressaceae being on average −24.6 mUr more depleted in ^2^H than the Pinaceae family (Figure 8c). Variations between different angiosperm orders were smaller, ranging from −160 to −219 mUr. Except of *I. aquifolium* which showed the least negative and *C. lawsoniana* the most negative fractionation of −139 and −243 mUr, respectively. For *Ginkgo biloba* L. the fractionation was equal to the median of gymnosperms. The mean ε_cell/xw_ values for angiosperm were 10.8 ± 15.8 mUr and for gymnosperm −4.9 ± 17.4 mUr. Variability within the two seed types was similar, although Pinales species can also be separated into more depleted Cupressaceae than Pinaceae species (Figure 8d). While most angiosperm species showed a positive ε_cell/xw_, most gymnosperm species showed negative ε_cell/xw_ values. The highest ε_cell/xw_ values were found in the orders Laminales, followed by Aquifliales and Saxifragales.

## 4 Discussion

### 4.1 Phylogenetic Pattern in Stable Isotopes of Methoxy Groups

Our study reveals phylogenetic patterns among leaf and twig *δ* ^2^H_meth_ and *δ* ^13^C_meth_ values, which were higher in leaves than in twigs (*δ* ^2^H_meth_: λ = 0.73 vs. 0.62, *δ* ^13^C_meth_: λ = 0.79 vs. 0.47) and primarily different at the seed type level between angiosperm and gymnosperms (Table 1, Figures 2, 3). In the following, we discuss reasons for our observation.

#### 4.1.1 Phylogeny in Hydrogen Isotopes of Methoxy Groups

Seed type‐specific variations which were seen in both *δ* ^2^H_meth_leaf_ and *δ* ^2^H_meth_twig_ values could be attributed to (A) temporal or spatial differences in the water uptake of the plants or (B) differences in plant metabolism, such as evolutionary adaptions that could cause differences in isotope fractionation. Regarding (A), most of our analysed gymnosperms are evergreen (16 out of 20) and the majority of angiosperms are deciduous (42 out of 46). Evergreen species can assimilate throughout the entire year, enabling them to photosynthesise also outside of the growing season (e.g., during early spring and autumn/winter) and use the isotopic composition of water sources that are different from those of the main growing season. In contrast, deciduous trees only assimilate during the growing season, limiting their isotopic signals to that period. For instance, the local *δ* ^2^H_precip_ values at our common garden site ranged from −74.5 mUr outside the growing season (October–March) to −40 mUr in the growing season (May–August) (averaged over the years 2017–2019; PISO AI; Nelson et al. [2021], Figure S2). Consequently, seasonal variability in water uptake of angiosperms is restricted to periods of ^2^H‐enriched summer precipitation, whereas gymnosperm species may exhibit greater variations in their water uptake period, reflecting a more ^2^H‐depleted annual precipitation signal. However, spatial variations in the water uptake, such as differences in the rooting depth can likely be exclude as *δ* ^2^H values of twig xylem and leaf water were not significantly different between angiosperms and gymnosperms at the sampling date (Schuler et al. 2023). Therefore large isotopic differences between angiosperms and gymnosperms (B) might also be explained by variations in the *δ* ^2^H of precursors (NADH, serine), and/or due to a varying degree of H‐exchange between organic compound and water and kinetic isotope effects in the plant C1 metabolic pathway leading to the biosynthesis of methoxy groups (Jardine et al. 2024; Greule et al. 2021; Holloway‐Phillips et al. 2022; Baan et al. 2023; Schuler et al. 2023; Lehmann et al. 2024). For example, Schuler et al. (2023) proposed a mutation in a gene for hypothetical protein essential for the ^2^H fractionation process during photosynthesis, explaining the evolutionary divergence between angiosperms and gymnosperms. However, some other studies suggested that the processes related to leaf shedding behaviour may explain differences in ^2^H‐fractionation (Arosio et al. 2020a, 2020b), however, the impact of leaf shedding in deciduous compared to evergreen conifer and angiosperms species, did not show any significant differences in *δ* ^2^H_meth_ values (*p* > 0.05) (Figure S3).

While *δ* ^2^H_meth_twig_ values showed no significant variation within seed types, *δ* ^2^H_meth_leaf_ values revealed additional phylogenetic differences within angiosperm species (λ = 0.79) (Table 1, Figure 2). This variation may be linked to the fact that, unlike woody material where the pectin content is much lower, leaves can contain significantly higher amounts of pectin (ranging between 7% and 35% of the cell wall material, Keppler et al. [2004]). We therefore removed the esterified methoxy groups of pectin from a subset of the sample material (49 species) following the method proposed by Cox, Laceby, et al. (2024) and Cox, Wieland, et al. (2024) and analysed the *δ* ^2^H_meth_leaf_ values of the remaining etherified methoxy groups of lignin. Lignin and pectin and only lignin‐related *δ* ^2^H_meth_leaf_ values differ in the same range, and no significant differences in the phylogenetic signals could be observed (λ = 0.73 and λ = 0.74) (Figure S4). Therefore, we can exclude pectin content as a main driver for *δ* ^2^H_meth_leaf_ variations between species in our study and further separation between the two compounds is not necessary to explain the species dependency in *δ* ^2^H_meth_ values. However, *δ* ^2^H_meth_leaf_ variations within angiosperm species might therefore be influenced by species‐specific differences in sources (such as a mixture of different serine pathways) and processes during lignin methoxy group biosynthesis.

#### 4.1.2 Phylogeny in Carbon Isotopes of Methoxy Groups

We found strong phylogenetic signals in *δ* ^13^C_meth_leaf_ values within both angiosperm families and between angiosperms and gymnosperms. These species‐specific variations were strongly reduced in the *δ* ^13^C_meth_twig_ material, where only the gymnosperm Cupressaceae family showed higher values compared to all other families (Table 2, Figure 3). The *δ* ^13^C values of plant material and compounds are mainly influenced by *δ* ^13^C of atmospheric CO_2_ (*δ* ^13^CO_2_), the isotope fractionation through the stomata and during carboxylation (discrimination by Rubisco), and by the inner leaf to ambient air CO_2_ concentration ratio (ci/ca) (Francey and Farquhar 1982). However, since the isotope fractionation due to air diffusion through the stomata and during carboxylation is considered to be constant, and the ambient CO_2_ concentration and *δ* ^13^CO_2_ levels are likely similar across the different species in our common garden experiment, we assume that the observed variations in *δ* ^13^C_meth_ values are primarily driven by different responses in leaf‐gas exchange influencing ci via changes in stomatal conductance and CO_2_ assimilation rate as a response to the environmental conditions in the common garden.

Higher *δ* ^13^C values in gymnosperms compared to angiosperms have been documented across various plant compounds including whole wood, lipids and cellulose in leaves and in cellulose tree rings (Leavitt and Newberry 1992; Diefendorf et al. 2010, 2012; Hare and Lavergne 2021). The differences were found to be likely attributed to the less efficient water transport system in conifers compared to broad‐leaved species, which affects hydraulic conductivity and, consequently, stomatal conductance (Lloyd et al. 1992; McCarroll and Loader 2004). Furthermore, Hare and Lavergne (2021) used a model to explore how differences in ^13^C fractionation between angiosperms and gymnosperms arise. They demonstrated that physiological factors, such as the higher carboxylation‐to‐transpiration ratio in angiosperms, lower mesophyll‐to‐stomatal conductance ratio in gymnosperms, and reduced CO_2_ release per oxygenation reaction during photorespiration in gymnosperms, lead to higher ^13^C fractionation and therefore more depleted *δ* ^13^C values in angiosperms compared to gymnosperms. Our observations showed significant species variation within angiosperm *δ* ^13^C_meth_leaf_ values, suggesting that seed type‐specific factors like stomatal conductance, photorespiration, or secondary cell wall composition also contribute to intra‐species differences among angiosperms. In gymnosperms, notable offsets in *δ* ^13^C_meth_leaf_ values were observed in deciduous species such as *L. kaempferi* and *M. glyptostroboides*. Conversely, some evergreen angiosperms like *Q. ilex* and *M. grandiflora* displayed *δ* ^13^C_meth_leaf_ values comparable to the gymnosperm average, implicating that the differences between angiosperms and gymnosperms are more likely related to their evergreen or deciduous traits. Next to the physiological differences, the wood nanostructure and the cell wall composition have been found to differ between angiosperm and gymnosperm species (Lyczakowski et al. 2019; Terrett and Dupree 2019; Lyczakowski and Wightman 2024). It was found that gymnosperms are characterised by large microfibrils of tracheids, while angiosperms showed small microfibrils of vessels. Differences in the cell wall assembly and composition might thereby impacting carbon sequestration and storage (Lyczakowski and Wightman 2024), which could further affect the *δ* ^13^C values of plant compounds.

In conclusion, seed type‐specific variances in *δ* ^2^H_meth_ seem to be attributed to temporal differences in water uptake or from variations in methoxy group biosynthesis, such as differing fractionation processes, precursors, or exchange mechanisms. Seed‐type‐specific differences in *δ* ^13^C_meth_ are likely attributed to variations in stomatal conductance, photorespiration, or secondary cell wall composition.

### 4.2 Biochemical Explanations for Leaf‐Twig Isotopic Pattern

*δ* ^13^C_meth_ values showed a strong offset between leaf and twig material (mean ΔC_leaf‐twig_ = −25.6 ± 6.6 mUr) with angiosperms (ΔC_leaf‐twig_: −27.8 ± 5.8 mUr) showing a stronger and more variating ΔC_leaf‐twig_ value than gymnosperm (ΔC_leaf‐twig_: −19.1 ± 3.7 mUr). Similar patterns were found in the study by Cox, Wieland, et al. (2024) finding *δ* ^13^C_meth_ values of litter being about 20 mUr lower compared to aboveground woody material (twigs and branches). However, such a ^13^C enrichment of non‐photosynthetic compared to photosynthetic tissues has been documented in several studies investigating bulk and carbohydrate *δ* ^13^C measurements (Cernusak et al. 2009; Bögelein et al. 2019; Rinne‐Garmston et al. 2023), suggesting and overall biochemical origin for this pattern. For example, the study of Rinne‐Garmston et al. (2023), found that *δ* ^13^C values of sugar have been progressively increased during the transport from leaves to roots and that glucose in phloem and roots is relatively enriched in ^13^C compared to sucrose. The authors concluded that the main cause of the observed ^13^C enrichment is associated with plant respiratory processes. In heterotrophic tissues, the pentose phosphate pathway causes dark‐respired CO_2_ to be ^13^C depleted relative to its substrate. Conversely, in leaves, the produced CO_2_ is assumed to be enriched in ^13^C, leaving ^13^C‐depleted carbon to be incorporated into leaf biomass (Cernusak et al. 2009; Bathellier et al. 2017; Rinne‐Garmston et al. 2023). In total, 410 studies analysing the isotopic composition of plant organs reported that leaves were on average, 1.26 ± 0.07 mUr more depleted in ^13^C compared to other plant organs (Badeck et al. 2005). However, the significantly larger ΔC_leaf‐twig_ observed in this study suggests that an additional process may be involved in methoxy group synthesis. To better understand this, it is important to note that a similar effect was not seen in ΔH_leaf‐twig_.

Lignin as a structural biomolecule is not considered to be transported through the vascular system (Boerjan et al. 2003), whereby the precursors of the OCH_3_ group (e.g., serine) have been found to be transported and synthesised within different plant tissues (Ros et al. 2014; Wang et al. 2024). The large offset seen in *δ* ^13^C_meth_ values between leaves and twig material but not in *δ* ^2^H_meth_ can be associated with different serine sources coupled with different synthesis pathways. As non‐foliar CO_2_ fixation is thought to occur with minimal photorespiration due to the high CO_2_ concentrations in woody tissues (Cernusak and Marshall 2000), the glycerate and glycolate pathways, which are coupled to photorespiration, may play little to no role, leaving PPLP as the primary serine source in woody tissues. This serine could have a different isotopic signature, influenced by its synthesis or by the contribution of respired CO_2_ during non‐foliar CO_2_ fixation. The reason we do not see a clear expression of the isotope effects in the PLPP for ΔH_leaf‐twig_, might be related to the likely strong isotopically depleted third hydrogen atom of the methyl group, transferred from NADH (isotopic signature down to −600 mUr compared to source water, Greule et al. [2021]), which dilutes the potential differences seen in serine. Thus, the NADH sources appear to be isotopically similar in the leaf and twig material in our study. However, it needs to be considered that the NADH source may vary in other woody parts of the plant further away from foliar tissues, as it was seen in root *δ* ^2^H_meth_ values in the study of Cox, Wieland, et al. (2024).

Interestingly, the *δ* ^2^H_meth_leaf_ values do not reflect the ^2^H enrichment in leaf water compared to twig xylem water, as observed by Schuler et al. (2023). This discrepancy might be explained by the varying *δ* ^2^H values of water within a leaf, where *δ* ^2^H values increase from the mid‐vein toward the leaf tip (Bariac et al. 1989; Šantrůček et al. 2007; Cernusak et al. 2016). For example, Šantrůček et al. (2007) reported a longitudinal *δ* ^2^H gradient (along the leaf midrib) of more than 80 mUr in arid‐grown Eucalyptus leaves. However, no significant *δ* ^2^H_meth_leaf_ enrichment compared to *δ* ^2^H_meth_twig_ values even when *δ* ^2^H_leaf_water_ was found to be enriched let suggest, that methoxy group precursor synthesis likely occurs closer to the leaf base or mid‐vein, as it was proposed for cellulose and *n*‐alkanes (Gamarra et al. 2016; Lehmann et al. 2017). Consequently, *δ* ^2^H_meth_leaf_ values appear to be more closely associated with xylem water. In conclusion, significant variances in *δ* ^13^C_meth_ and the absence of notable differences in *δ* ^2^H_meth_ values between heterotrophic and autotrophic material implicated that the precursors of methoxy groups partly differ across plant tissues. While the NADH source appears consistent between leaves and twigs, serine biosynthesis may vary. In leaves, serine is likely derived from the glycerate, glycolate and PPLP pathways, whereas in heterotrophic tissues, it is primarily supplied by the PPLP. In addition, the lack of *δ* ^2^H_meth_leaf_ differences implicated that leaf water enrichment processes do not influence methoxy synthesis, indicating that *δ* ^2^H_meth_ primarily reflects xylem water variations.

### 4.3 Apparent Isotopic Fractionation Between Water and Methoxy Groups

The mean ε_meth/xw_ value in this study was found to be −197 mUr, considering both seed types equally. The phylogenetic signal λ in ε_meth/xw_ was similar to that in *δ* ^2^H_meth_twig_ values (λ = 0.64 and 0.62) indicating that the *δ* ^2^H_xw_ values are not the main driver for the phylogenetic pattern in the methoxy groups. The mean ε_meth/xw_ of this study is similar to that calculated in the study by Greule et al. (2021), where an overall isotope fractionation of around −200 mUr between annual *δ* ^2^H_precip_ and *δ* ^2^H_meth_ values was determined (ε_meth/precip_) across a range of *δ* ^2^H_precip_ from −20 to −110 mUr. Porter et al. (2022) separated the seed types angiosperm and gymnosperms measured in Greule et al. (2021) and found significant differences between ε_meth/precip_, with gymnosperms being more depleted than angiosperms (−204 ± 12 and −196 ± 14 mUr, respectively). Similar to the observations of Porter et al. (2022), we found that ε_meth/xw_ values significantly differed between angiosperm and gymnosperm species (*p* < 0.001), with gymnosperm ε_meth/xw_ values being on average 26 mUr lower than angiosperms. Moreover, Anhäuser, Greule and Keppler (2017) analysed the ε_meth/precip_ from four different tree species including angiosperms and gymnosperms, using modelled annual *δ* ^2^H_precip_ data across 15 German study sites. Comparison between the results of Anhäuser, Greule and Keppler (2017) and this study showed that ε_meth/precip_ differed between seed type but are consistent across several sample sites. For instance, ε_meth/precip_ for *P. abies* and *Q. robur* were −214 and −190 mUr in Anhäuser, Greule and Keppler (2017) and −210 and −184 mUr in this study. In the case of cellulose, the apparent fractionation (ε_cell/xw_) was around 0 and thus similar to results across northern hemisphere trees normalised to precipitation isotopes (Lehmann et al. 2022), however, also show strong differences between gymnosperms (16 mUr lower) and angiosperms (Figure 8).

**Figure 9:**
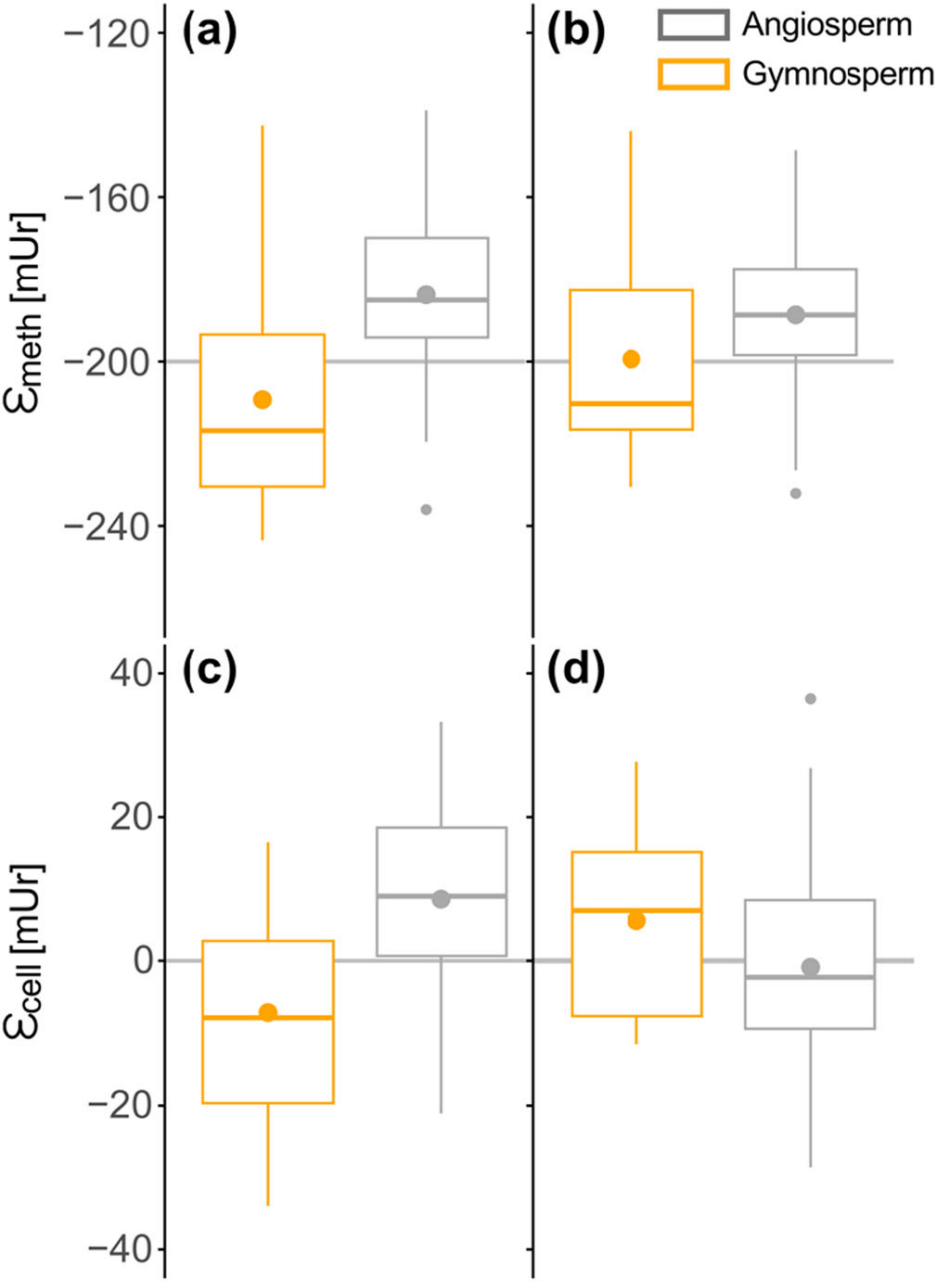
Seed type‐specific hydrogen isotope fractionation between (a) twig methoxy groups and xylem water, and (b) between twig methoxy groups and annual precipitation for gymnosperms (orange) and summer precipitation (May to August) for angiosperms (grey). The horizontal grey solid lines in (a) and (b) represent ε_meth/precip_ of −200 mUr by Greule et al. (2021). (c) Hydrogen isotope fractionation between twig cellulose and xylem water and (d) between twig cellulose and precipitation, with the same precipitation references as in panel (b). The grey solid lines in (c) and (d) represent no isotope fractionation (0). In all plots, gymnosperms are coloured orange and angiosperms in grey, and the boxplots showing the mean (points), and median (horizontal line) values with whiskers represent the 95% CI. [Color figure can be viewed at wileyonlinelibrary.com]

The seed type difference in the apparent isotope fractionation of both compounds might be partially explained by the *δ* ^2^H_xw_ values used for isotope fractionation calculation (Figure 9a,c), which were only sampled once in the end of August 2019. However, *δ* ^2^H_xw_ values can greatly differ over the year due to strong variations in the *δ* ^2^H_precip_ values and therefore the tree source water. As previously mentioned, gymnosperms are mostly evergreen species and can assimilate throughout the whole year, whereas most angiosperms are deciduous trees assimilating during the growing season. We therefore calculated isotope fractionations between the *δ* ^2^H_meth_twig,_ *δ* ^2^H_cell_twig_ and modelled *δ* ^2^H_precip_ values (PISO AI, Nelson et al. [2021]) (ε_meth/precip_) using precipitation from May to August for angiosperms and annual means (2017–2019) for gymnosperms (Figure 9b,d). The seed type‐specific isotope fractionations are now closer to each other with ε_meth/precip_ values of −189 ± 18.5 mUr for angiosperms and −199.4 ± 23.3 mUr for gymnosperms and ε_cell/precip_ values of −0.9 ± 14.4 mUr for angiosperms and 7.4 ± 14.5 mUr for gymnosperms, supporting the assumption that deciduous tree species use precipitation restricted from the growing season, while evergreen species represent isotopic annual precipitation signatures.

### 4.4 Comparison Between Methoxy Groups and Carbohydrates

The hydrogen isotope fractionation between xylem water and methoxy groups is significantly higher compared to that xylem water and cellulose, likely due to the highly ^2^H‐depleted third hydrogen from NADH, incorporated into the methyl group during C1 metabolism and methoxy group synthesis (Figure 8). Interestingly, species‐specific variations in *δ* ^2^H_meth_twig_ and *δ* ^2^H_cell_twig_ values showed little correlation with only 9%–14% of the variations in methoxy groups attributable to carbohydrates. That these variations are connected to the different years represented in the *δ* ^2^H_meth_twig_ (average of the years 2017–2019) and *δ* ^2^H_cell_twig_ (simply 2019) values seems unlikely, as mean *δ* ^2^H_precip_ values were consistent over these 3 years (May–August −40 to −47 mUr, January to December −57.5 to −62.9 in mUr, Nelson et al. [2021]). Previous studies have documented a strong temporal correlation between the *δ* ^13^C values of cellulose and methoxy groups in tree rings, however, correlation between *δ* ^2^H_meth_ and *δ* ^2^H_cell_ values was found to be weaker (Römer et al. 2025; Gori et al. 2013; Mischel et al. 2015; Wieland et al. 2024). Similar *δ* ^13^C response in both compounds (Gori et al. 2013; Mischel et al. 2015; Schmidt et al. 2015; Wieland et al. 2024) might arise from the similar precursor of carbon as cellulose consists of β‐1,4 linked glucose chains and the C6 position of glucose is also the main source of C3 serine, the primary source of the C1 unit further used for methoxy group synthesis. In contrast, the difference in the *δ* ^2^H response between cellulose and methoxy groups is likely explained by differences in the metabolic pathways. *δ* ^2^H_meth_ values most likely originate from only two distinct biochemical pathways (Greule et al. 2021), resulting in fewer enzymatic effects and hydrogen source variations compared to cellulose (Holloway‐Phillips et al. 2025).

Beside isotope fractionation processes within metabolic pathways, temporal differences in lignin and carbohydrate biosynthesis could also lead to distinct *δ* ^2^H patterns. Lignification, for instance, occurs in the final stages of xylem, cell differentiation, where lignin is deposited within the carbohydrate matric of cell walls (Bogolitsyn et al. 2021). If the formation of these compounds occurs at different times during the growing season, variations in water sources could further contribute to these distinct isotope patterns.

## 5 Conclusion

Our hydrogen and carbon isotope measurements of methoxy groups from leaf and twig material revealed significant differences between seed types as well as pronounced inter‐species variability in leaf material among angiosperms. Seed type‐specific *δ* ^2^H_meth_ variations are likely driven by temporal plant water uptake variations or evolutionary divergences of angiosperms and gymnosperms resulting in differences in ^2^H fractionation, whereas variations of *δ* ^13^C_meth_ may result from differences in stomatal conductance, CO_2_ assimilation rate, or secondary cell wall composition between evergreen and deciduous trees. In addition, we found strong offsets between leaf and twig *δ* ^13^C_meth_ but not in *δ* ^2^H_meth_ values (−25.6 ± 6.6 and +3.4 ± 16.3 mUr, respectively), suggesting that precursors, such as serine, are not simply transported through the vascular system and instead are additionally synthesised through the PPLP in heterotrophic plant tissues. However, NADH which provides the third and likely exceptionally ^2^H‐depleted hydrogen atom for the precursor of the methoxy group, seems to originate from the same source in twigs and leaves, diluting potential differences in *δ* ^2^H of serine from different pathways. Hydrogen isotopic fractionation between tree xylem water and twig methoxy groups showed strong differences between the seed types, with mean apparent fractionation of gymnosperm being −209 ± 25.9 mUr and angiosperm −184 ± 18.7 mUr. A comparison with twig *δ* ^2^H values of carbohydrates from the same trees showed that methoxy groups are significantly more depleted than cellulose and both compounds show only little coherence. As *δ* ^2^H of methoxy groups likely originates from only two distinct biochemical pathways, fewer competing enzymatic effects and hydrogen sources are combined compared to *δ* ^2^H of carbohydrates. Furthermore, as lignin is one of the final stages in the differentiation of xylem cells, different water sources or temporal variations in the biosynthesis of carbohydrates and lignin might cause different isotope pattern along the biosynthetic pathways, indicating that their combined use could serve as a powerful complementary tool in future studies. For example, to identify seasonal root water uptake or metabolic changes. For further stable isotope methoxy group analysis including both leaf and twig material, it is essential to consider phylogenetic differences between angiosperm and gymnosperm species. However, it is not necessary to consider species‐specific variations in twig methoxy groups within seed types, as these differences were negligible.

## Conflicts of Interest

The authors declare no conflicts of interest.

## Supporting information


**Figure S1:** Pagels l of (a) *δ* ^2^Hmeth_leaf, (b) *δ* ^2^Hmeth_twig, (c) *δ* ^13^Cmeth_leaf, and (d) *δ* ^13^Cmeth_twig. **Figure S2:** Monthly variations of *δ* ^2^Hprecip values from the years 2017‐2019, data received from Piso Ai (Nelson et al. 2021). **Figure S3:** Violine plots of hydrogen (*δ* ^2^Hmeth,) (a, c) and carbon (*δ* ^13^Cmeth) (b, d) isotope ratios of leaf (a, b) and twig (c, d) methoxy groups across individuals of 64 trees and shrubs species. In all panels angiosperms are colored grey and gymnosperm orange, deciduous trees in lighter and evergreen trees in the darker color, respectively. Significant differences (*p* < 0.05) are represented with different letter (compact letter display). The boxplots within the violin plots are showing the mean (points) and median (horizontal line) values with whiskers representing the 95% CI. **Figure S4:** Phylogenetic tree showing hydrogen isotope ratios of lignin leaf methoxy groups. l shows Pagel's l used to estimate the phylogenetic signal, with corresponding *p*‐value for significance estimation.

## Data Availability

The data that support the findings of this study is available online at https://doi.org/10.16904/envidat.640 (Lehmann et al. 2025).
