# Supplementary material for: Dual Isotope Analysis Reveals Phylogenetic Patterns and Novel Insights Into Methoxy Group Synthesis of Structural Biomolecules in Leaf and Woody Plant Tissues
Source: Plant Cell Environ. 2025 Aug 25;48(12):8483–501. doi: 10.1111/pce.70134 (PMC12586915; doi:10.1111/pce.70134)
Supplement: Supplementary file 1 — Figure S1: Pagels l of (a) δ 2Hmeth_leaf, (b) δ 2Hmeth_twig, (c) δ 13Cmeth_leaf, and (d) δ 13Cmeth_twig. Figure S2: Monthly variations of δ 2Hprecip values from the years 2017‐2019, data received from Piso Ai (Nelson et al. 2021). Figure S3: Violine plots of hydrogen (δ 2Hmeth,) (a, c) and carbon (δ 13Cmeth) (b, d) isotope ratios of leaf (a, b) and twig (c, d) methoxy groups across individuals of 64 trees and shrubs species. In all panels angiosperms are colored grey and gymnosperm orange, deciduous trees in lighter and evergreen trees in the darker color, respectively. Significant differences (p < 0.05) are represented with different letter (compact letter display). The boxplots within the violin plots are showing the mean (points) and median (horizontal line) values with whiskers representing the 95% CI. Figure S4: Phylogenetic tree showing hydrogen isotope ratios of lignin leaf methoxy groups. l shows Pagel's l used to estimate the phylogenetic signal, with corresponding p‐value for significance estimation. [file PCE-48-8483-s001.docx]

**Supplemental material**

Article title: Dual isotope analysis reveals phylogenetic patterns and novel insights into methoxy group synthesis of structural biomolecules in leaf and woody plant tissues

Authors: Anna Wieland, Philipp Schuler, Matthias Saurer, Valentina Vitali, Markus Greule, Frank Keppler, and Marco M. Lehmann

Article acceptance date: 09 August 2025


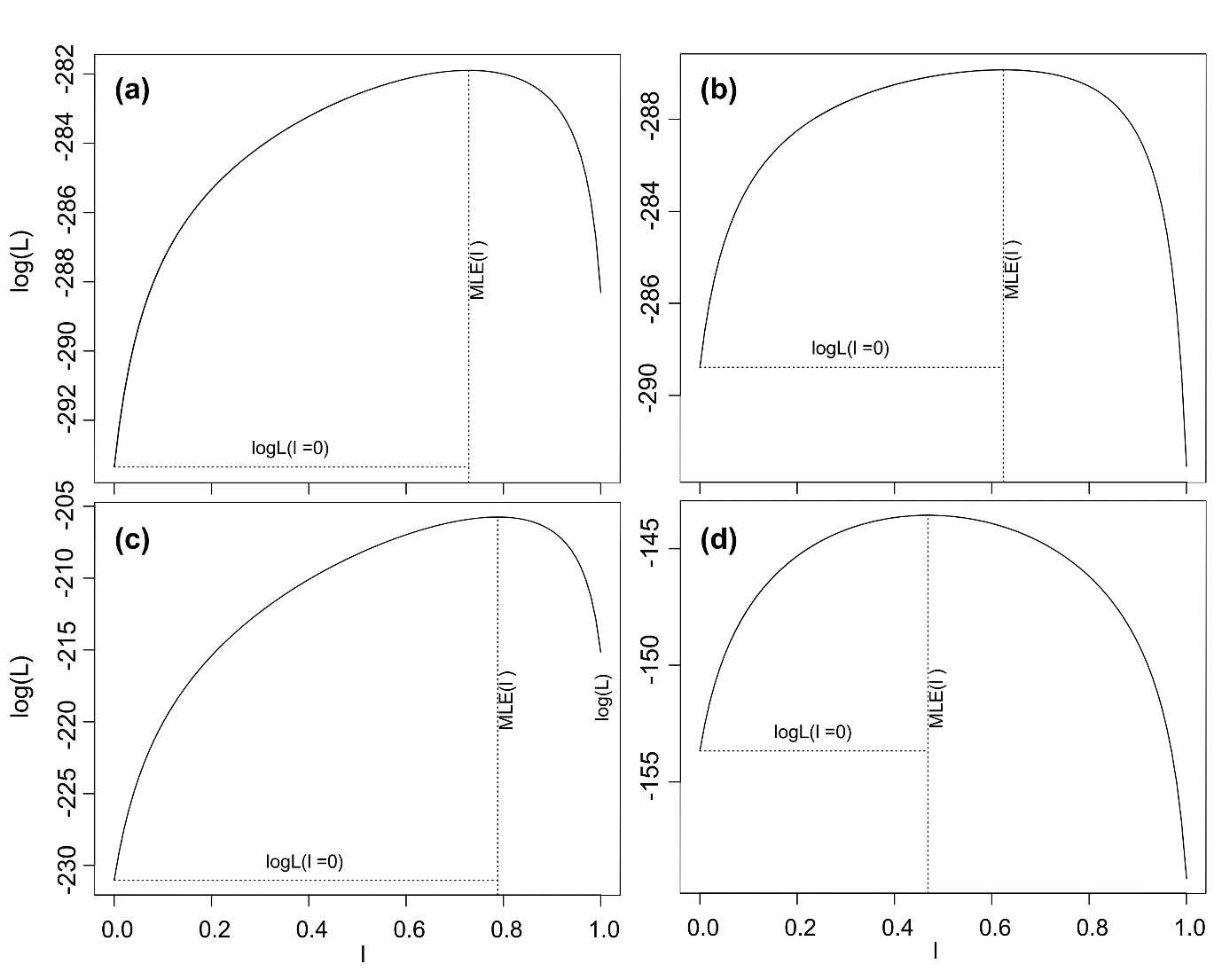


**S1**. Pagels λ of (a) *δ*²H_meth_leaf_, (b) *δ*²H_meth_twig_, (c) *δ*^13^C_meth_leaf_, and (d) *δ*^13^C_meth_twig_.


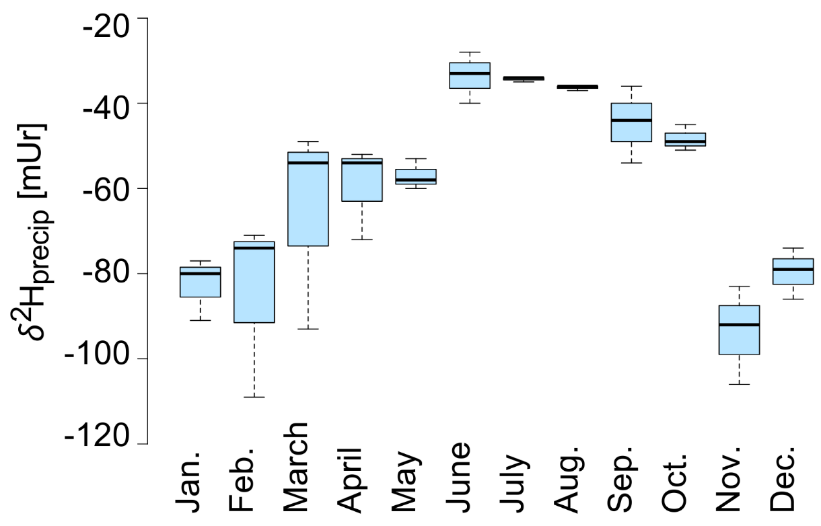


**S2.** Monthly variations of *δ*²H_precip_ values from the years 2017-2019, data received from Piso Ai (Nelson et al. 2021).


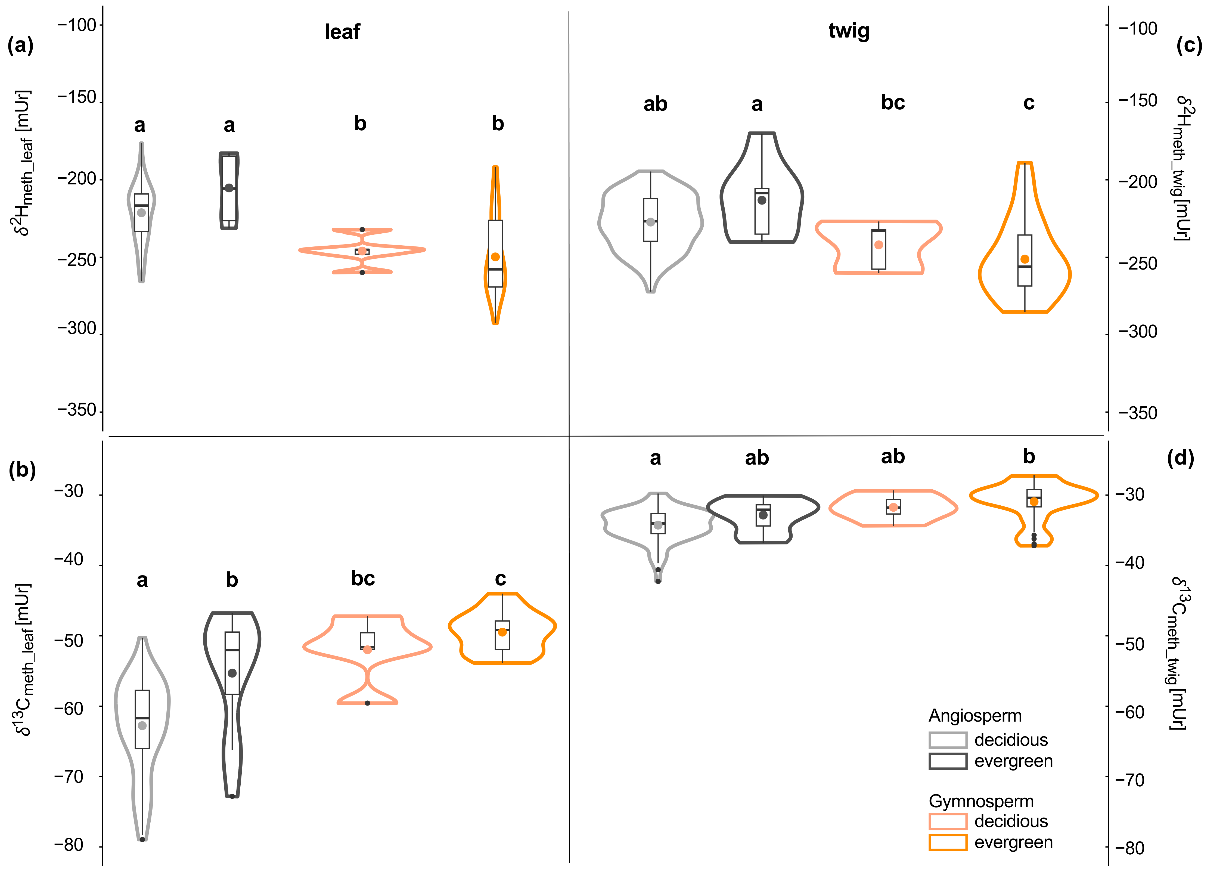


**S3.** Violine plots of hydrogen (*δ*²H_meth_,) (a, c) and carbon (*δ*^13^C_meth_) (b, d) isotope ratios of leaf (a, b) and twig (c, d) methoxy groups across individuals of 64 trees and shrubs species. In all panels angiosperms are colored grey and gymnosperm orange, deciduous trees in lighter and evergreen trees in the darker color, respectively. Significant differences (p < 0.05) are represented with different letter (compact letter display). The boxplots within the violin plots are showing the mean (points) and median (horizontal line) values with whiskers representing the 95 % CI.


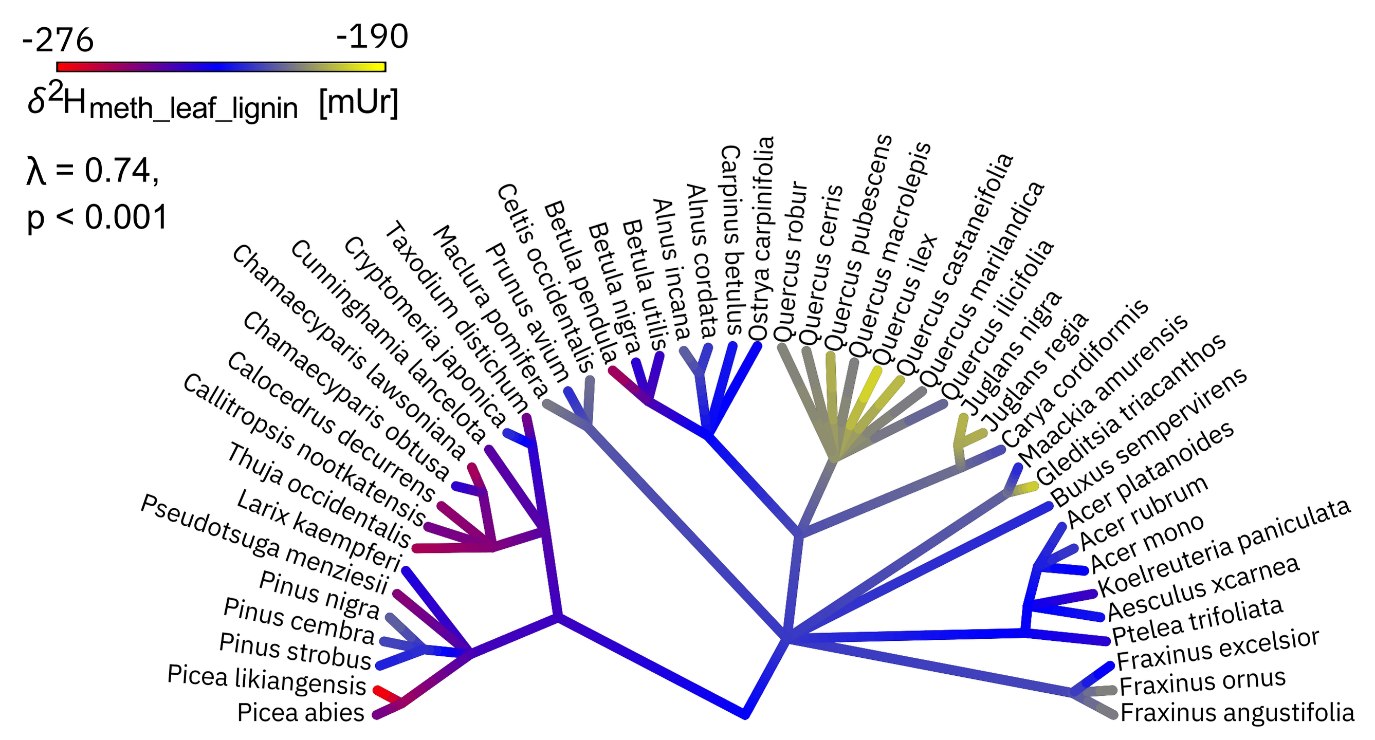


**S4**. Phylogenetic tree showing hydrogen isotope ratios of lignin leaf methoxy groups. λ shows Pagel’s λ used to estimate the phylogenetic signal, with corresponding p-value for significance estimation.
